# Is it worth it? Cost-effectiveness analysis of a commercial physical activity app

**DOI:** 10.1186/s12889-021-11988-y

**Published:** 2021-10-27

**Authors:** Renante Rondina, Michael Hong, Sisira Sarma, Marc Mitchell

**Affiliations:** 1 Rotman School of Management, University of Toronto, Toronto, ON Canada; 2 Schulich School of Medicine and Dentistry, Western University, London, ON Canada; 3 Faculty of Health Sciences, Western University, London, ON Canada

**Keywords:** Cost-effectiveness, mHealth, Public health, Physical activity, Behavioural economics

## Abstract

**Background:**

Government interest in investing in commercial physical activity apps has increased with little evidence of their cost-effectiveness. This is the first study to our knowledge to examine the cost-effectiveness of a commercial physical activity app (*Carrot Rewards*) despite there being over 100,000 in the major app stores.

**Methods:**

A cost-effectiveness analysis was performed to calculate the incremental cost-effectiveness ratio (ICER) of the app compared to a no-intervention reference scenario using a five-year time horizon. Primary data was collected between 2016 and 2017. Data synthesis, model creation, and statistical analyses were conducted between 2019 and 2020. An age-, sex-, and geography-dependent Markov model was developed assuming a public healthcare payer perspective. A closed cohort (*n* = 38,452) representing the population reached by *Carrot Rewards* in two Canadian provinces (British Columbia, Newfoundland & Labrador) at the time of a 12-month prospective study was used. Costs and effects were both discounted at 1.5% and expressed in 2015 Canadian dollars. Subgroup analyses were conducted to compare ICERs between provinces, sexes, age groups, and engagement levels.

**Results:**

*Carrot Rewards* had an ICER of $11,113 CAD per quality adjusted life year (QALY), well below a $50,000 CAD per QALY willingness-to-pay (WTP) threshold. Subgroup analyses revealed that the app had lower ICERs for British Columbians, females, highly engaged users, and adults aged 35-64 yrs., and was dominant for older adults (65 + yrs). Deterministic sensitivity analyses revealed that the ICER was most influenced by the relative risk of diabetes. Probabilistic sensitivity analyses revealed varying parameter estimates predominantly resulted in ICERs below the WTP threshold.

**Conclusions:**

The *Carrot Rewards* app was cost-effective, and dominant for older adults. These results provide, for the first time, rigorous health economic evidence for a commercial physical activity app as part of public health programming.

**Supplementary Information:**

The online version contains supplementary material available at 10.1186/s12889-021-11988-y.

## Background

Physical inactivity is the most prevalent modifiable chronic disease risk factor with about 85 % of adults worldwide not meeting recommended physical activity guidelines [1–4]. The case for population-level intervention is increasingly based on the costs of physical inactivity [5, 6]. In response to recent evidence of the societal cost of physical inactivity (globally $53.8 billion USD), [6] the World Health Organization (WHO) set bold physical inactivity reduction targets—15% relative reduction in the global prevalence of insufficient physical activity in adults and adolescents by 2030 [7]. The WHO singled out digital innovation (e.g., mobile health applications, or mHealth apps) as an important component of a broad “systems-based” solution in their *Global Action Plan on Physical Activity* [7]. Despite their apparent potential, and growing interest amongst governments [8–10] and corporations, [11, 12] a small number of commercial physical activity apps have been independently evaluated in peer-reviewed journals (i.e. only about 15 out of more than 100,000 in the major app stores) [13–16]. Existing evaluations have found mixed results, with some finding app-based interventions increase physical activity, [13, 14] while others report no effect [15, 17]. Most importantly, no cost-effectiveness analysis of *any* commercial physical activity app has to our knowledge been published to date. In this study, we evaluate the cost-effectiveness of a commercial physical activity app, Carrot Rewards, which was found to increase objectively-measured daily step counts in a 12-month prospective cohort study.

Evidence standards for digital health technologies suggest that in addition to establishing the relevance, acceptability and effectiveness of mHealth apps in increasing physical activity, it is imperative to recognize their economic impact [18–20]. Cost-effectiveness studies facilitate public health policy decision making, allowing skilled policy-makers to compare interventions in terms of costs and effects and determine whether funding is justified in fiscally constrained environments [20–22]. Despite the proliferation of physical activity apps, which has accelerated since the WHO declared COVID-19 a global pandemic on March 11, 2020, [23] the lack of cost-effectiveness has been cited as a major barrier to policy investment (i.e. government investment in physical activity apps) [18, 20, 24]. A 2017 systematic review of economic evaluations of mHealth solutions in general uncovered 39 studies that largely reported positive economic outcomes (e.g., increase in life years gained, cost savings) [19]. Only nine of the included studies used an app as the mHealth function, and among those none targeted physical activity. The positive outcomes from this review must be interpreted with caution, however, given the lack of rigour in many of the included studies identified by the review authors (e.g., incomplete economic evaluations, short intervention periods, no sensitivity analyses, etc.). The widespread design deficiencies make it difficult to respond to the common criticism that cost-effectiveness is often assumed, without evidence to support it [19].

To address this issue, cost-effectiveness analyses of ‘top tier’ commercial apps should be prioritized given their mass appeal (i.e. the top 2% reporting more than 500,000 monthly active users (MAUs) [25]). Unlike previous cost-effectiveness analyses of physical activity interventions more broadly (outside the mHealth context e.g., mass media campaigns), [26–28] analyses should model risk reductions based on *objectively-measured* (vs. subjective measures) and *longer-term* (6+ months, the theoretical threshold for behaviour maintenance) [29] changes in physical activity [19, 20, 30]. Age- and sex-specific models should also be used since disease incidence and mortality rates vary widely by demographic group [19, 30, 31]. To further minimize bias, sensitivity analyses are needed to evaluate variables that most influence results [19, 20, 30]. Lastly, while randomized controlled trials (RCTs) are preferred data sources for cost-effectiveness studies, they are notoriously difficult to conduct in fast-paced commercial digital environments. Iribarren et al. (2017) and others suggest that prospective cohort studies, especially longitudinal ones, may also provide high quality data for cost-effectiveness studies of mHealth interventions [19, 20].

Financial health incentive programs continue to be popular with 56% of large U.S. employers (and at least 15% of European employers), [12] for instance, offering rewards worth $946 USD per year to employees for participating in healthy activities [11]. While a concern with financial incentives is that they can be prohibitively costly, [32] technological advances have made tracking and rewarding physical activity easier and more immediate. This, combined with stronger application of behaviour change theory, has driven the cost of rewards down to pennies a day potentially increasing cost-effectiveness [33]. Carrot Rewards was a ‘top tier’ commercial physical activity app available in Canada only (i.e. 1.3+ million downloads, 500,000+ MAUs as of May 2019) [34]. It leveraged gamification elements and concepts from behavioural economics and self-determination theory to reward users with very small ($0.04 CAD) financial incentives (i.e. points redeemable for consumer goods) to walk more [32]. Our objective, therefore, is to conduct a cost-effectiveness analysis of a ‘top tier’ commercial physical activity app that uses financial incentives to drive healthy behaviour.

## Methods

A cost-effectiveness analysis was performed to estimate the relative costs and effects of the Carrot Rewards app compared to a reference scenario where no intervention was available. We developed an age-, sex-, and geography-dependent Markov model assuming a public healthcare payer perspective, given Carrot Rewards was initially publicly funded. All costs are in 2015 Canadian dollars, and both costs and quality adjusted life years (QALYs) are discounted at 1.5% per year, as recommended by the Canadian Agency for Drugs and Technologies in Health [35]. A Consolidated Health Economic Evaluation Reporting Standards (CHEERS) checklist was completed [30]. Additional details regarding the cohort and data sources are in the Additional File 1. Ethical approval for this study was provided by Western University’s Human Research Ethics Board (#113322). This study involved the secondary use of de-identified data. Therefore, the need for informed consent was waived for this secondary data analysis by Western University’s Human Research Ethics Board. All methods were carried out in accordance with the relevant guidelines and regulations. App users were informed of and had to accept the app’s privacy policy describing how de-identified data may be used for reporting purposes and presented in aggregate.

### Cohort

The model uses a closed cohort representing the population reached by the intervention at the time of the study (*n* = 38,452). The cohort from which the data was collected from were users who completed the download and registration of the app during the recruitment period between June 13th to July 10th, 2016. Data from this cohort was collected for 12 months between 2016 and 2017. The target population was female and male youth (13 to 17 years) and adults (18+ years) living in two Canadians provinces: British Columbia and Newfoundland & Labrador (the first two to fund the app for their constituents). App users were classified into four engagement groups: ‘Limited’ (fewer than 12 weeks in which the app was used at least once), ‘Occasional’ (12–23 weeks), ‘Regular’ (24–51 weeks), or ‘Committed’ (52 weeks) (Additional File 2).

### Model design

To model improvement in physical activity, we will use daily step counts collected using built-in smartphone accelerometers and reported in a 12-month prospective cohort evaluation of the Carrot Rewards app [31]. Step counts will be linked to chronic disease risk reductions from available databases. The model was developed in 2019 and is presented in Additional File 3. It assumes all cohort members start in a health state free of events, and consists of five chronic diseases with well-established associations with physical inactivity, [36, 37] although we acknowledge new evidence is accumulating that supports the inverse relationship between physical activity and more than 20 other chronic conditions (e.g., depression, bladder cancer, osteoporosis) [38]. Health states comprised: (i) healthy; (ii) ischaemic heart disease (IHD); (iii) stroke; (iv) diabetes mellitus; (v) colorectal cancer; (vi) breast cancer; and (vii) death. We assumed a five-year time horizon and a cycle length of one year. At the end of each cycle, individuals had an annual probability of either remaining in the same health state or transitioning into a different one. Transitions between health states were allowed once per cycle. Individuals in a chronic disease state either remained in the same chronic disease state or transitioned to death. They could not progress backwards to the healthy state and could not have co-morbid conditions.

### Data sources

Transition probabilities were based on annual incidence and mortality rates reported in Additional Files 4 and 5, respectively. The data from which these rates were based upon came from Statistics Canada [39] and Canadian Chronic Disease Surveillance System [40] database, and are therefore representative of the actual subgroups. The extent to which Carrot Rewards increased daily step count was drawn from the 12-month cohort study (i.e. 448.8 and 884.6 step per day increase for ‘Regular’ and ‘Committed’ users, respectively) [31] and converted into a standardized energy expenditure metric using the formula from Wu et al. (2000) [41]. Recent meta-analyses of step count monitoring [42] and physical activity incentive interventions [33] report similar daily step count improvements at 6-to-12 months (i.e. 670 and 464–1050 steps/d, respectively). We assumed daily step count increases noted at 12-months would be sustained each year over the five-year time horizon since improvements beyond six months are considered stable [29, 42]. Relative risks are presented in Additional File 6 [43–55] for ‘Regular’ and ‘Committed’ users only, as the 12-month study showed no improvements in ‘Limited’ and ‘Occasional’ users. Relative risks reflect the improvement in transition probabilities from a healthy state to a diseased state due to physical activity. The studies from which these relative risks were based upon drew from a population of similarly aged male and female youths and adults from similar geographic regions, and we assumed a direct linear relationship between physical activity and risk reduction.

Only direct medical costs were considered (i.e. drugs, physician care, and hospital care). The average annual medical cost for each of the five chronic diseases are presented in Additional File 7 The data from which these costs were based upon came from the Economic Burden of Illness in Canada [56] database, and are therefore representative of the actual subgroups.

The cost of the Carrot Rewards app was based exclusively on the amount spent on loyalty points to reward all users for registering on the app ($0.60), meeting daily step goals ($0.04/day), and completing weekly step challenges ($0.40 if users reached their step goal 10 non-consecutive times in a 2-week period). Intervention costs were paid for by government partners. We assumed individuals had to continue participating in order to maintain the daily step count increase. Additional File 8 presents the estimated annual cost of the intervention based on the 12-month data. For health-related quality of life, utility data as measured by the EQ-5D were obtained from the literature and reported in Additional File 9 [57–61]. Data were retrieved in 2018 and synthesized in 2019.

### Sensitivity analyses

To capture uncertainty associated with these parameters, deterministic sensitivity analyses were performed by individually varying each parameter, and a probabilistic sensitivity analysis (Monte Carlo) was performed by varying all parameters concurrently. Transition probabilities between health states followed a beta distribution, risk ratios followed a log-normal distribution, and intervention costs followed a gamma distribution [62]. Analyses were completed in 2020.

## Results

### Base case

For the no-intervention arm the average discounted QALY amounted to 4.6348 with a cost of $113.16. Implementing Carrot Rewards improved the average QALY by 0.0011 to 4.6359 and increased the cost by $11.86 to $125.02, for an ICER of $11,113.31/QALY. Subgroup analyses are presented in Table 1. The intervention had a lower ICER for: (a) females ($7959.82/QALY) versus males ($15,896.01/QALY), (b) British Columbia ($9945.20/QALY) versus Newfoundland & Labrador ($14,239.54/QALY), (c) ‘Committed’ ($2715.39/QALY) versus ‘Regular’ users ($14,583.77/QALY), and (d) older versus younger users (13–19, $80,376.60/QALY, 20–34: $32,602.45/QALY; 35–49: $4062.39/QALY; 50–64: $7516.79/QALY; 65–79: dominant).

**Table 1:** Subgroup analyses by province, gender, engagement level, and age

	Costs (CAD $)			Effectiveness (QALYs)			ICER
	Standard	Carrot	Incremental	Standard	Carrot	Incremental	
**Province**							
BC	$ 106.46	$ 117.08	$ 10.62	4.6369	4.6380	0.0011	$ 9945.20
NL	$ 131.02	$ 146.16	$ 15.14	4.6291	4.6302	0.0011	$ 14,239.54
**Gender**							
Female	$ 104.55	$ 112.19	$ 7.64	4.6565	4.6575	0.0010	$ 7959.82
Male	$ 130.63	$ 151.03	$ 20.40	4.5907	4.5920	0.0013	$ 15,896.01
**Engagement**							
Regular	$ 101.94	$ 119.71	$ 17.77	4.6417	4.6429	0.0012	$ 14,583.77
Committed	$ 160.92	$ 168.64	$ 7.72	4.5937	4.5965	0.0028	$ 2715.39
**Age Group (years)**							
13–19	$ 7.51	$ 24.51	$ 17.00	4.7264	4.7266	0.0002	$ 80,376.60
20–34	$ 31.38	$ 48.70	$ 17.32	4.7018	4.7023	0.0005	$ 32,602.45
35–49	$ 163.23	$ 169.67	$ 6.44	4.6384	4.640	0.0016	$ 4062.39
50–64	$ 384.78	$ 401.08	$ 16.30	4.3215	4.3236	0.0021	$ 7516.79
65+	$ 853.94	$ 674.98	$ (178.96)	3.6698	3.6779	0.0081	dominant

*QALYs* quality adjusted life years.

*ICER* incremental cost-effectiveness ratio.

*BC* British Columbia.

*NL* Newfoundland & Labrador

### Sensitivity analyses

**Fig. 1:**
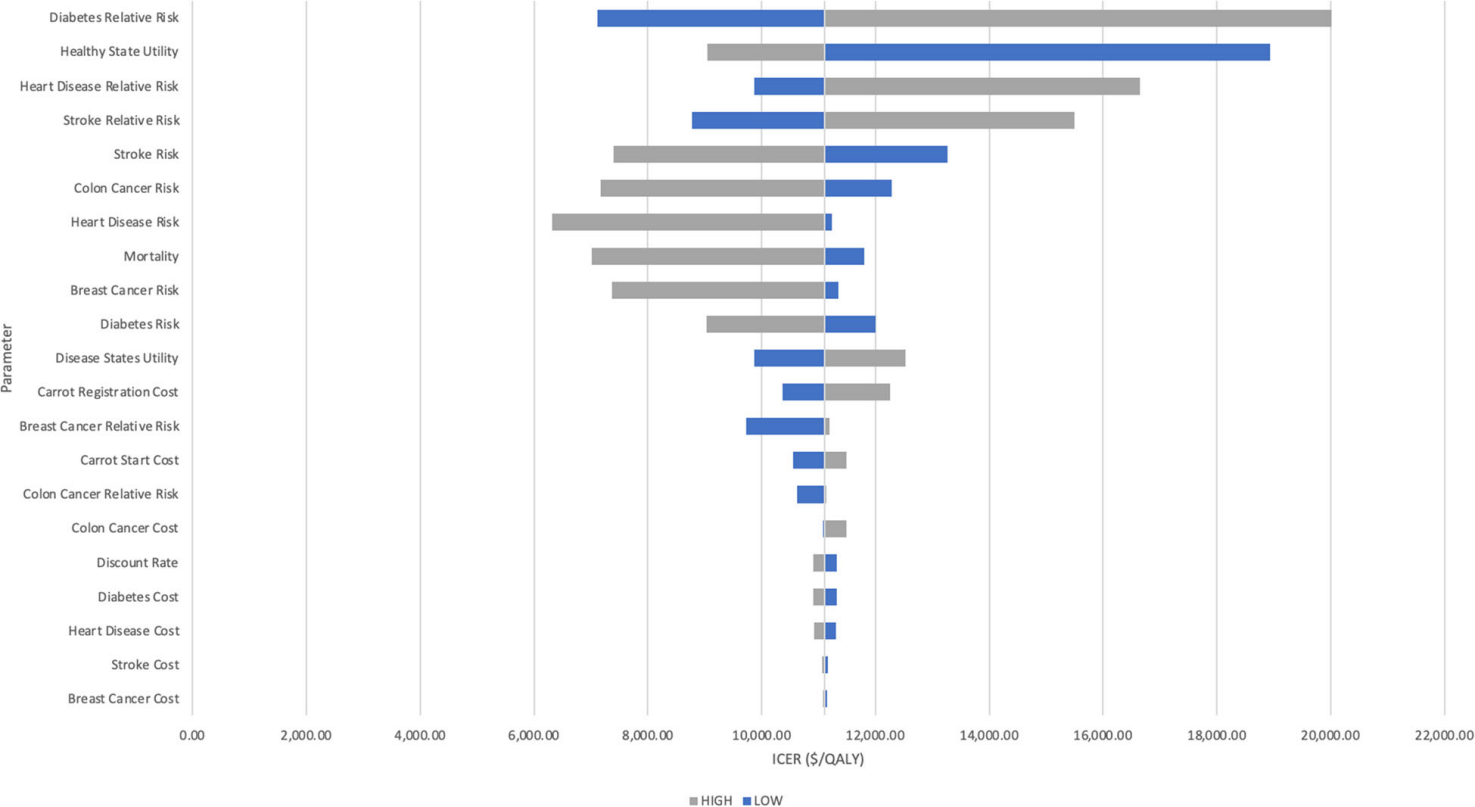
Tornado diagram for deterministic sensitivity analyses. ICER: incremental cost-effectiveness ratio. High/Low: parameter set to the upper (grey) or lower (blue) bound of the confidence interval

**Fig. 2:**
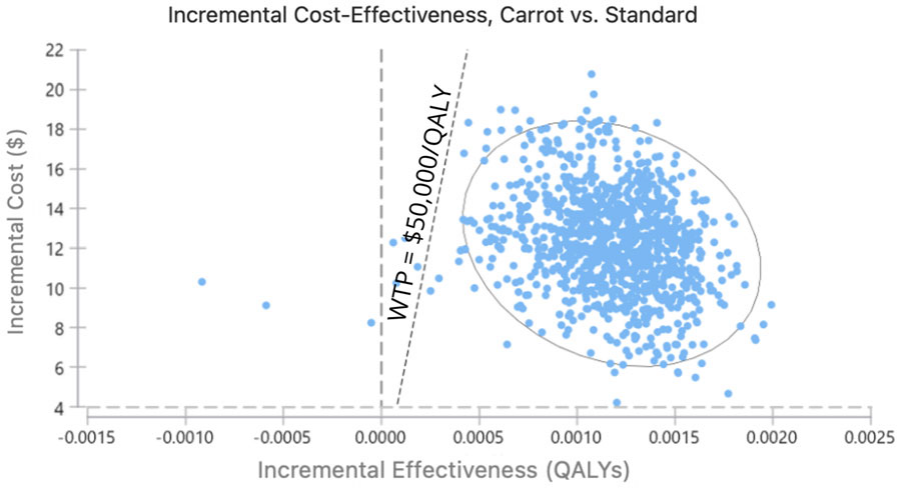
Probabilistic sensitivity analyses based on 10,000 Monte Carlo simulations. WTP: willingness-to-pay. Grey ellipse represents 95% confidence intervals

**Fig. 3:**
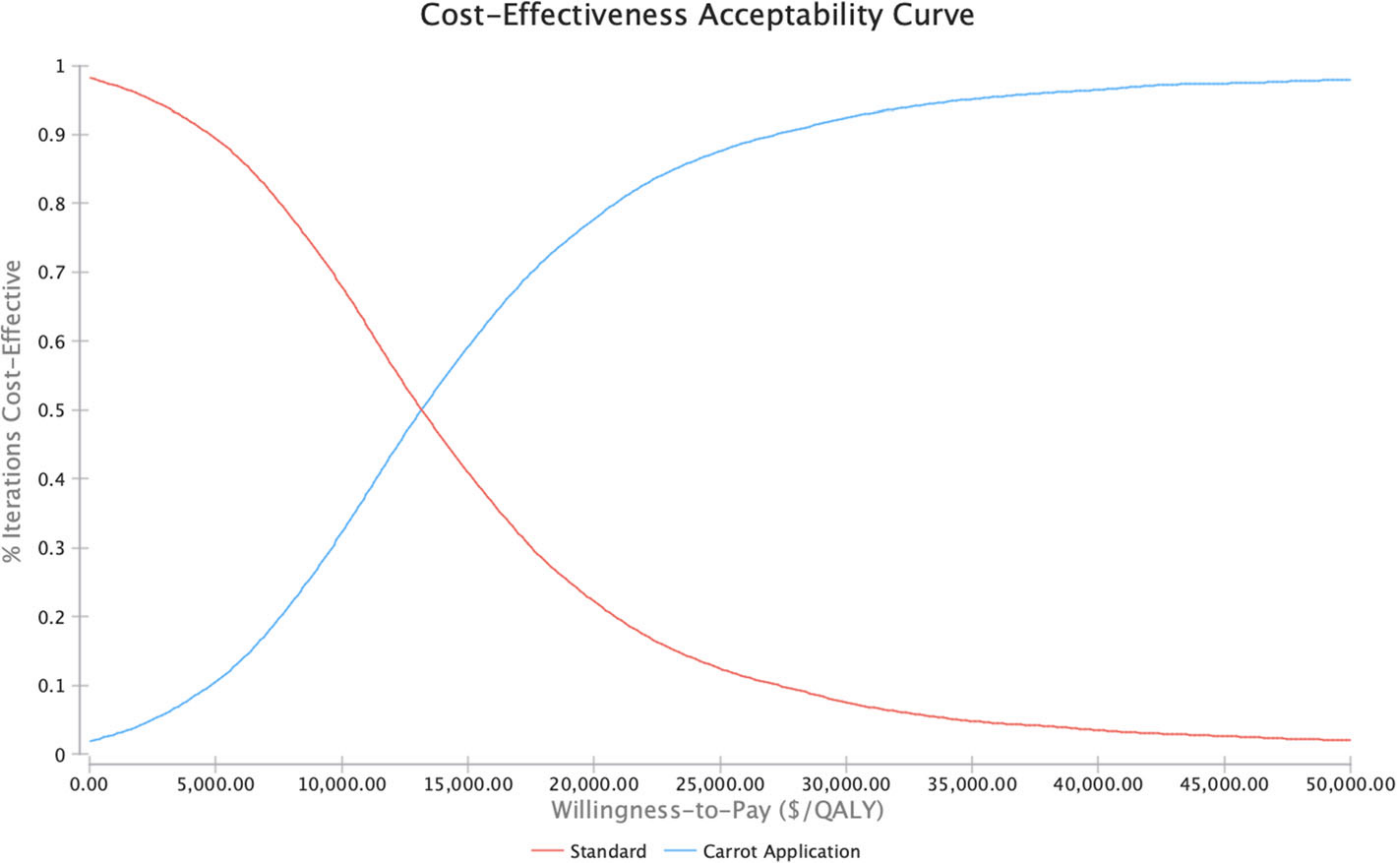
Cost-effectiveness acceptability curve

The influence of each parameter is demonstrated in Fig. 1 with larger bars having a greater influence on the variation in the model (also see Additional File 10). The parameter with the largest influence was the relative risk for diabetes with an upper bound of $20,015.08. The findings of the probabilistic sensitivity analysis are shown in Fig. 2. Based on 10,000 simulations, Carrot Rewards was more effective than no-intervention more than 99% of the time and cost more than no-intervention 100% of the time. Figure 3 shows that Carrot Rewards surpassed the no-intervention scenario at a willingness-to-pay (WTP) of $10,386.26.

## Discussion

### Main finding

This is the first study to evaluate the cost-effectiveness of a commercial physical activity app despite there being over 100,000 published in major app stores. We found Carrot Rewards was cost-effective over a five-year time horizon relative to an arbitrary WTP threshold of $50,000/QALY ($11,113.31/QALY). For comparison to economic benchmarks, the Canadian Gross Domestic Product per capita is about $60,000 [63]. These results are relevant for countries with publicly-funded healthcare systems (e.g., Canada, U.K., Germany, Australia) but also corporations considering mHealth apps that target employee physical inactivity given the short- (e.g., depression management) and long-term (e.g., type 2 diabetes risk reduction) benefits of physical activity [38]. Until now, policy-makers were not able to compare traditional physical activity interventions (e.g., mass media campaigns, pedometer interventions) with newer mHealth approaches in terms of costs and effects. This study begins to answer the question ‘Are commercial physical activity apps cost-effective?’ and may help policy-makers determine whether funding is justified in light of some of our parameters (e.g., 449–885 steps/day expected intervention effect, intervention cost of $4–$11/year). Specifically, Carrot Rewards produced physical activity increases with incentives that were at least 50 times smaller than what has been used in previous research (e.g., $0.04 vs. $2.00 *per day*) [33] and corporate settings (e.g., $4–$11 vs. $1247 *per year*) [11].

Recent evidence suggests that reward size may be less important than other incentive intervention design features (e.g., incentive timing or form) [64]. It has been suggested that manipulating these other features (outlined by Adams et al. [65] and updated by Mitchell et al. [66]) may help reduce the cost of incentives while maintaining or increasing effects [33]. The small incentives used by Carrot Rewards increased physical activity, in part, because they were offered *immediately*, thereby exploiting the behavioural economic concept of “present bias”, which is the human tendency to prefer payoffs close to the present [67, 68]. This and other theoretically-informed manipulations may appeal to governments and corporations looking to deploy physical activity incentives as efficiently as possible [33]. Others researchers have demonstrated positive effects with physical activity incentives worth $0.09 to $0.75 USD per day when implemented as part of a multicomponent physical activity intervention [69–72].

### Secondary findings

Carrot Rewards had a lower ICER in British Columbia than Newfoundland & Labrador, possibly explained by higher engagement levels in British Columbia. As well, the app’s ICER was two times lower in females than males and was cost-effective for all age groups over 20 years and *dominant* over 65 years. The larger effects by age are due to higher baseline rates of chronic conditions as age increases, leading to a greater number of chronic conditions prevented. By sex, although there is a larger incremental effect by males, there is also a larger incremental cost by males, this is due to a smaller reduction in costs related to chronic conditions among males and a larger cost associated with the Carrot application. Carrot Rewards’s ICER was five-times lower in users who engaged for 52 weeks versus those who engaged less often. Notably, in this study, ‘Limited’ and ‘Occasional’ app users incurred costs without benefit. Deterministic sensitivity analyses revealed that estimated cost-effectiveness was most influenced by the relative risk of diabetes. The probabilistic sensitivity analysis revealed that varying parameter estimates across a wide range of uncertainty mostly resulted in ICERs below the $50,000 WTP threshold, and a small number of iterations resulted in ICERs above this threshold. Taken together, our results suggest that an mHealth app with incentives may be most cost-effective for working aged (20 to 64 yrs) and older (65 + yrs) females. As well, continued efforts to maximize app engagement (e.g., with regular behavioural science-informed feature upgrades) [15] and minimize reward magnitudes (e.g., by weaning users off daily incentives after 3–4 months) [33] may yield greater cost-effectiveness.

### Related studies

Beyond the dearth of cost-effectiveness evaluations of commercial physical activity apps, a few related studies help put our results in context. Cost-effectiveness evaluations of physical activity interventions in general have mostly determined that pedometer-based interventions are most cost-effective in Australia, Belgium and the Netherlands (with ICERs ranging from €11,100/QALY to dominant) [73–75]. For example, one pedometer-based intervention reported an estimated cost-savings of €500 per person and 0.11–0.16 QALYs gained (more favorable than reported for Carrot Rewards) [74]. Similarly, workplace physical activity incentive programmes have proved to be cost-effective in the UK (with ICERs ranging from not cost-effective to £2900/QALY) [76, 77]. For example, a cost-effectiveness study of a workplace physical activity incentive programme reported estimated incremental costs of £4100 and 1.2 QALYs gained [77]. The considerable disparity in study designs generally makes it difficult to draw direct comparisons to the present study. For instance, the current mHealth intervention was delivered on a population-scale (vs. similar studies that base predictions on pilot data), [74] used an objectively-measured physical activity outcome (vs. similar studies that used change estimates from separate meta-analyses, or self-report), [73–75] and had a younger sample (vs. older samples in similar studies which increases cost-effectiveness) [74, 78] making head-to-head comparisons difficult. We should note that while Carrot Rewards had a relatively small impact on overall QALYs, the impact was greater amongst ‘Committed’ and older users. This is somewhat comparable to previous studies who reported QALY increases of 0.16 and 0.11 for males and females, respectively, but who used a longer time horizon and whose risk reductions were not based on objectively-measured step count increases.^58^

### Limitations and future directions

We made multiple conservative decisions in how our model was structured. First, given that death was a possible outcome, our decision to employ a 5-year time horizon may omit longer term consequences. Extending the horizon may lead to more favorable ICERs. Second, our decision to not include co-morbidities may have also led to less favorable ICERs, as co-morbidities would increase medical costs while decreasing quality of life. Therefore, the positive effects of physical activity delaying disease onset would have produced more favorable results. Finally, we did not include all chronic diseases associated with physical inactivity (e.g., mood and anxiety disorders) [79]. The inclusion of these diseases and their physical activity-related risk reductions into the model would have resulted in more favorable ICERs. This is notable as 27.3% of Carrot Rewards users self-reported a physician diagnosed mood and anxiety disorder after the current study period (unpublished). Therefore, since we assumed all users started in a healthy state, we may have underestimated cost-effectiveness. As we were not able to link participants’ diagnoses with their objectively-measured physical activity, future studies should also consider the health and economic outcomes of similar interventions stratified by health status. We also made some assumptions to convert objectively-measured daily step count increases to risk reductions. First, we assumed a direct linear relationship between physical activity and risk reduction when in fact it is curvilinear with greater risk reduction at lower initial doses (e.g., going from 0 to 30 min as opposed to from 120 to 150 min). This suggests that our model may be underestimating risk reduction and cost-effectiveness given the generally low baseline physical activity levels of our cohort (i.e. 43% accumulated less than 5000 steps per day) [31]. Second, while our intervention was 12 months-long we assumed physical activity increases persisted for five years, as others have done with similar or shorter duration interventions [21, 73–75]. Third, while conservative, we assumed daily step count increases occurred at the lower end of moderate intensity [38] which may not have been the case. Future economic evaluations of accelerometer-based interventions should also take differences in physical activity intensity into consideration when estimating effects on risk reduction.

We also took the perspective of a publicly funded healthcare system as rewards were funded by government partners. A societal perspective would have more favorable ICERs. Our model also includes programmatic costs of the financial incentives only. The model does not include company overhead, as company overhead was funded through other (non-government) revenue sources, or development costs as they were viewed as sunk costs—not normally included as they have no impact on the marginal costs and benefits of continuing an intervention [80].

## Conclusions

This is the first cost-effectiveness study of a commercially available physical activity app. We have shown that an incentive-based mHealth app targeting physical activity would be cost-effective in two Canadian provinces over a five-year time horizon. As digital health technologies continue to evolve to address emerging and persistent global health issues, it is incumbent on researchers and policy-makers to demand a broader evidence-base that includes health economic impacts to inform public health policy decisions.

## Supplementary Information

**Additional file 1.** Detailed description of methods, cohort, and data sources.

**Additional file 2.** Cohort composition by age, gender, geography, and engagement level.

**Additional file 3.** Illustration of Markov model design: in this state-transition diagram all of the seven health states are inserted. Each arrow is linked with a certain transition probability. Circles represent possible health states. The following states can be distinguished: healthy, diabetes, colorectal cancer, breast cancer, (ischemic) heart disease, stroke, and death.

**Additional file 4.** Age-, gender-, and geography-dependent transition probabilities by chronic disease.

**Additional file 5.** Age-, gender-, and disease-dependent mortality rates by health state.

**Additional file 6.** Relative risk by engagement level, age group, and gender.

**Additional file 7.** Age-, gender-, and geography dependent average annual medical costs by chronic disease.

**Additional file 8.** Age-, gender-, and geography-dependent average annual intervention costs by engagement level.

**Additional file 9.** Age-, gender-, and geography dependent average annual medical costs by chronic disease.

**Additional file 10.** Lower and upper bounds for each parameter in the deterministic sensitivity analysis.

## Data Availability

The datasets used and/or analysed during the current study are available from the corresponding author on reasonable request.

## Abbreviations

WHO: World Health Organization
ICER: Incremental cost-effectiveness ratio
MAU: Monthly active user
mHealth: Mobile health
RCT: Randomized controlled trial
CAD: Canadian dollars
USD: U.S. dollars
QALY: Quality-adjusted life years
CHEERS: Consolidated Health Economic Evaluation Reporting Standards
IHD: Ischemic heart disease
EQ-5D: EuroQol 5D
