# Supplementary material for: Is it worth it? Cost-effectiveness analysis of a commercial physical activity app
Source: BMC Public Health. 2021 Oct 27;21:1950. doi: 10.1186/s12889-021-11988-y (PMC8548862; doi:10.1186/s12889-021-11988-y)
Supplement: Supplementary file 1 — Additional file 1. Detailed description of methods, cohort, and data sources. [file 12889_2021_11988_MOESM1_ESM.docx]

**Additional File 1:** Detailed description of methods, cohort, and data sources.

**Cohort**

Following a two-week baseline period, app users earned digital incentives ($0.04 CAD) every day they reached a personalized daily step goal. Mixed-effects models estimated changes in weekly mean daily step count between the baseline period and the last two recorded weeks. Models were fit for several engagement groups.

**Data Sources**

Regarding risk reductions, the 12-month prospective cohort study (Mitchell et al., 2020) showed an increase of 3,141.6 steps per week for ‘Regular’ users and 6,192.2 steps per week for ‘Committed’ users. These were converted into MET-hrs per week using the formula from Wu et al. (2000). An increase of 1.34 and 2.63 MET-hrs per week were calculated for ‘Regular’ and ‘Committed’ users, respectively. A linear relationship was assumed between MET-hr increases and risk reductions in order to calculate the estimate relative risk for these two engagement groups. Step count increases were not observed among app users engaging for fewer than 24 weeks.

The average annual medical cost for each of the seven chronic diseases by age, gender, and province were estimated by dividing the total medical costs of each chronic disease for each province reported by PHAC’s 2015 Economic Burden of Illness in Canada (EBIC) by the prevalence rate of each disease reported by PHAC’s 2015 CCDSS and the available literature (Ellison & Wilkins, 2009). The EBIC only reported costs for 15- to 34-year-olds, so similar costs were estimated for the 13- to 19-year-old group and the 20- to 34-year-old group. The EBIC did not report costs of breast cancer in men, so costs for women were used for both gender groups. The EBIC also did not report costs for Type 1 and Type 2 diabetes separately, so costs for both types were used for the diabetes health state.
