# Supplementary material for: Is it worth it? Cost-effectiveness analysis of a commercial physical activity app
Source: BMC Public Health. 2021 Oct 27;21:1950. doi: 10.1186/s12889-021-11988-y (PMC8548862; doi:10.1186/s12889-021-11988-y)
Supplement: Supplementary file 2 — Additional file 2. Cohort composition by age, gender, geography, and engagement level. [file 12889_2021_11988_MOESM2_ESM.docx]

**Additional File 2:** Cohort composition by age, gender, geography, and engagement level.

|  | **British Columbia** | | **Newfoundland & Labrador** | |
| --- | --- | --- | --- | --- |
|  | **Female** | **Male** | **Female** | **Male** |
| **Limited Users** |  |  |  |  |
| 13-19 yrs | 284 | 211 | 119 | 63 |
| 20-34 | 1882 | 1141 | 1032 | 369 |
| 35-49 | 966 | 505 | 531 | 164 |
| 50-64 | 419 | 186 | 184 | 54 |
| 65-79 | 53 | 34 | 14 | 10 |
| **Occasional Users** |  |  |  |  |
| 13-19 | 231 | 133 | 96 | 40 |
| 20-34 | 1745 | 993 | 870 | 348 |
| 35-49 | 885 | 422 | 494 | 154 |
| 50-64 | 348 | 150 | 125 | 46 |
| 65-79 | 29 | 22 | 8 | 4 |
| **Regular Users** |  |  |  |  |
| 13-19 | 437 | 206 | 195 | 51 |
| 20-34 | 4011 | 2191 | 1586 | 497 |
| 35-49 | 2077 | 1118 | 942 | 294 |
| 50-64 | 790 | 344 | 287 | 92 |
| 65-79 | 71 | 57 | 15 | 15 |
| **Committed Users** |  |  |  |  |
| 13-19 | 193 | 88 | 84 | 27 |
| 20-34 | 1919 | 1064 | 590 | 296 |
| 35-49 | 1212 | 728 | 418 | 209 |
| 50-64 | 398 | 291 | 138 | 56 |
| 65-79 | 48 | 37 | 7 | 9 |

Limited: users engaged for fewer than 12 weeks

Occasional: users engaged for 12 to 23 weeks

Regular: users engaged for 24 to 51 weeks

Committed: users engaged for 52 weeks
