# Supplementary material for: Is it worth it? Cost-effectiveness analysis of a commercial physical activity app
Source: BMC Public Health. 2021 Oct 27;21:1950. doi: 10.1186/s12889-021-11988-y (PMC8548862; doi:10.1186/s12889-021-11988-y)

**Additional File 3.** Illustration of Markov model design: in this state-transition diagram all of the seven health states are inserted. Each arrow is linked with a certain transition probability. Circles represent possible health states. The following states can be distinguished: healthy, diabetes, colorectal cancer, breast cancer, (ischemic) heart disease, stroke, and death.


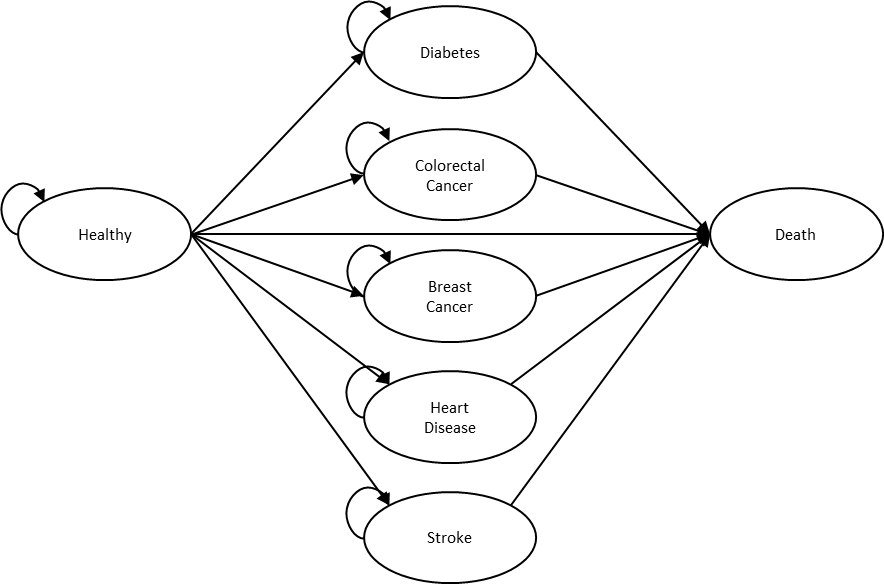

Supplement: Supplementary file 3 — Additional file 3. Illustration of Markov model design: in this state-transition diagram all of the seven health states are inserted. Each arrow is linked with a certain transition probability. Circles represent possible health states. The following states can be distinguished: healthy, diabetes, colorectal cancer, breast cancer, (ischemic) heart disease, stroke, and death. [file 12889_2021_11988_MOESM3_ESM.docx]
