# Supplementary material for: Is it worth it? Cost-effectiveness analysis of a commercial physical activity app
Source: BMC Public Health. 2021 Oct 27;21:1950. doi: 10.1186/s12889-021-11988-y (PMC8548862; doi:10.1186/s12889-021-11988-y)
Supplement: Supplementary file 4 — Additional file 4. Age-, gender-, and geography-dependent transition probabilities by chronic disease. [file 12889_2021_11988_MOESM4_ESM.docx]

**Additional File 4.** Age-, gender-, and geography-dependent transition probabilities by chronic disease.

| **Risk of Disease** | | | | |
| --- | --- | --- | --- | --- |
|  | Expected Value | 95% CI | Distribution | Reference |
| **Breast Cancer** |  |  |  |  |
| BC Female, 20-34 | 0.000138 | (0.000079, 0.000197) | Beta(21.01, 152252.52) | Statistics Canada (2016) |
| BC Female, 35-49 | 0.001056 | (0.000908, 0.001204) | Beta(195.37, 184813.02) | Statistics Canada (2016) |
| BC Female, 50-64 | 0.002268 | (0.002054, 0.002482) | Beta(430.51, 189388.31) | Statistics Canada (2016) |
| BC Female, 65-79 | 0.003714 | (0.003345, 0.004083) | Beta(387.72, 104007.63) | Statistics Canada (2016) |
| BC Female, 80+ | 0.003305 | (0.002742, 0.003869) | Beta(131.94, 39790.60) | Statistics Canada (2016) |
| BC Male, 50-64 | 0.000029 | (0.000003, 0.000055) | Beta(4.78, 164791.81) | Statistics Canada (2016) |
| BC Male, 65-79 | 0.000049 | (0.000011, 0.000087) | Beta(6.39, 130345.23) | Statistics Canada (2016) |
| BC Male, 80+ | 0.000173 | (0.000005, 0.000298) | Beta(4.07, 23538.08) | Statistics Canada (2016) |
| NL Female, 20-34 | 0.000318 | (0.000020, 0.000616) | Beta(4.37, 13746.71) | Statistics Canada (2016) |
| NL Female, 35-49 | 0.001284 | (0.000806, 0.001762) | Beta(27.68, 21532.04) | Statistics Canada (2016) |
| NL Female, 50-64 | 0.002736 | (0.002052, 0.003420) | Beta(61.29, 22341.74) | Statistics Canada (2016) |
| NL Female, 65-79 | 0.004104 | (0.002989, 0.005219) | Beta(51.83, 12576.60) | Statistics Canada (2016) |
| NL Female, 80+ | 0.003964 | (0.002119, 0.005809) | Beta(17.66, 4437.17) | Statistics Canada (2016) |
| NL Male, 50-64 | 0.000237 | (0.000034, 0.000439) | Beta(5.23, 22082.23) | Statistics Canada (2016) |
| **Colon Cancer** |  |  |  |  |
| BC Female, 20-34 | 0.000052 | (0.000014, 0.000089) | Beta(7.19, 138324.78) | Statistics Canada (2016) |
| BC Female, 35-49 | 0.000178 | (0.000115, 0.000240) | Beta(30.66, 172224.09) | Statistics Canada (2016) |
| BC Female, 50-64 | 0.000656 | (0.000543, 0.000770) | Beta(129.38, 197100.13) | Statistics Canada (2016) |
| BC Female, 65-79 | 0.001532 | (0.001284, 0.001780) | Beta(146.37, 95396.25) | Statistics Canada (2016) |
| BC Female, 80+ | 0.002833 | (0.002307, 0.003359) | Beta(111.12, 39112.17) | Statistics Canada (2016) |
| BC Male, 13-19 | 0.000036 | (0.000005, 0.000067) | Beta(5.18, 143898.73) | Statistics Canada (2016) |
| BC Male, 20-34 | 0.000045 | (0.000014, 0.000076) | Beta(8.09, 179870.43) | Statistics Canada (2016) |
| BC Male, 35-49 | 0.000181 | (0.000116, 0.000246) | Beta(29.78, 164514.48) | Statistics Canada (2016) |
| BC Male, 50-64 | 0.000487 | (0.000341, 0.000632) | Beta(42.72, 87681.37) | Statistics Canada (2016) |
| BC Male, 65-79 | 0.002336 | (0.002019, 0.002653) | Beta(208.12, 88885.35) | Statistics Canada (2016) |
| BC Male, 80+ | 0.003278 | (0.002561, 0.003994) | Beta(80.03, 24333.98) | Statistics Canada (2016) |
| NL Female, 35-49 | 0.000273 | (0.000033, 0.000514) | Beta(4.97, 18196.64) | Statistics Canada (2016) |
| NL Female, 50-64 | 0.001328 | (0.000879, 0.001778) | Beta(33.56, 25237.49) | Statistics Canada (2016) |
| NL Female, 65-79 | 0.002897 | (0.001857, 0.003936) | Beta(29.72, 10228.96) | Statistics Canada (2016) |
| NL Female, 80+ | 0.004360 | (0.002131, 0.006589) | Beta(14.63, 3340.83) | Statistics Canada (2016) |
| NL Male, 35-49 | 0.000388 | (0.000242, 0.000533) | Beta(27.12, 69870.66) | Statistics Canada (2016) |
| NL Male, 50-64 | 0.001672 | (0.001404, 0.001939) | Beta(149.27, 89129.29) | Statistics Canada (2016) |
| NL Male, 65-79 | 0.004357 | (0.003734, 0.004979) | Beta(187.07, 42748.49) | Statistics Canada (2016) |
| NL Male, 80+ | 0.006261 | (0.004605, 0.007918) | Beta(54.56, 8660.24) | Statistics Canada (2016) |
| **Diabetes** |  |  |  |  |
| BC Female, 13-19 | 0.00042 | (0.00036, 0.00048) | Beta(188.16, 447809.27) | CCDS (2015) |
| BC Female, 20-34 | 0.00155 | (0.00144, 0.00166) | Beta((761.58, 490580.44) | CCDS (2015) |
| BC Female, 35-49 | 0.00459 | (0.00440, 0.00478) | Beta(2231.68, 483972.55) | CCDS (2015) |
| BC Female, 50-64 | 0.00917 | (0.00890, 0.00944) | Beta(4390.58, 474407.26) | CCDS (2015) |
| BC Female, 65-79 | 0.01206 | (0.01164, 0.01249) | Beta(3056.03, 250346.19) | CCDS (2015) |
| BC Female, 80+ | 0.01125 | (0.01061, 0.01191) | Beta(1137.82, 100001.52) | CCDS (2015) |
| BC Male, 13-19 | 0.00044 | (0.00038, 0.00050) | Beta(206.50, 469114.79) | CCDS (2015) |
| BC Male, 20-34 | 0.00128 | (0.00118, 0.00139) | Beta(570.16, 444866.84) | CCDS (2015) |
| BC Male, 35-49 | 0.00580 | (0.00558, 0.00603) | Beta(2537.91, 435032.42) | CCDS (2015) |
| BC Male, 50-64 | 0.01276 | (0.01244, 0.01309) | Beta(5846.13, 452314.62) | CCDS (2015) |
| BC Male, 65-79 | 0.01820 | (0.01765, 0.01876) | Beta(4055.93, 218797.14) | CCDS (2015) |
| BC Male, 80+ | 0.01477 | (0.01571, 0.01388) | Beta(986.19, 65783.82) | CCDS (2015) |
| NL Female, 13-19 | 0.00042 | (0.00025, 0.00064) | Beta(17.81, 42395.24) | CCDS (2015) |
| NL Female, 20-34 | 0.00171 | (0.00136, 0.00213) | Beta(75.65, 44166.24) | CCDS (2015) |
| NL Female, 35-49 | 0.00641 | (0.00575, 0.00713) | Beta(329.40, 51059.67) | CCDS (2015) |
| NL Female, 50-64 | 0.00525 | (0.00470, 0.00585) | Beta(318.57, 60361.00) | CCDS (2015) |
| NL Female, 65-79 | 0.01713 | (0.01572, 0.01864) | Beta(519.76, 29822.32) | CCDS (2015) |
| NL Female, 80+ | 0.01528 | (0.01286, 0.01804) | Beta(131.65, 8484.19) | CCDS (2015) |
| NL Male, 13-19 | 0.00079 | (0.00056, 0.00107) | Beta(36.84, 46597.55) | CCDS (2015) |
| NL Male, 20-34 | 0.00149 | (0.00116, 0.00188) | Beta(65.71, 44034.03) | CCDS (2015) |
| NL Male, 35-49 | 0.00691 | (0.00622, 0.00767) | Beta(346.56, 49806.12) | CCDS (2015) |
| NL Male, 50-64 | 0.01575 | (0.01473, 0.01683) | Beta(850.73, 53163.89 | CCDS (2015) |
| NL Male, 65-79 | 0.02526 | (0.02346, 0.02716) | Beta(698.09, 26937.88) | CCDS (2015) |
| NL Male, 80+ | 0.01849 | (0.01519, 0.02228) | Beta(102.56, 5444.10) | CCDS (2015) |
| **Heart Disease** |  |  |  |  |
| BC Female, 20-34 | 0.00029 | (0.00024, 0.00034) | Beta(129.19, 445366.17) | CCDS (2015) |
| BC Female, 35-49 | 0.00137 | (0.00127, 0.00148) | Beta(653.10, 476060.79) | CCDS (2015) |
| BC Female, 50-64 | 0.00523 | (0.00503, 0.00543) | Beta(2613.23, 497047.99) | CCDS (2015) |
| BC Female, 65-79 | 0.01276 | (0.01235, 0.01318) | Beta(3585.41, 277402.61) | CCDS (2015) |
| BC Female, 80+ | 0.02238 | (0.02147, 0.02333) | Beta(2174.86, 95004.00) | CCDS (2015) |
| BC Male, 20-34 | 0.00048 | (0.00043, 0.00055) | Beta(245.74, 511720.73) | CCDS (2015) |
| BC Male, 35-49 | 0.00273 | (0.00258, 0.00288) | Beta(1269.01, 463571.62) | CCDS (2015) |
| BC Male, 50-64 | 0.00974 | (0.00946, 0.01002) | Beta(4603.23, 468007.25) | CCDS (2015) |
| BC Male, 65-79 | 0.02082 | (0.02025, 0.02141) | Beta(4847.06, 227960.73) | CCDS (2015) |
| BC Male, 80+ | 0.03051 | (0.02914, 0.03192) | Beta(1794.33, 57016.93) | CCDS (2015) |
| NL Female, 20-34 | 0.00042 | (0.00025, 0.00064) | Beta(17.81, 42395.24) | CCDS (2015) |
| NL Female, 35-49 | 0.00163 | (0.00131, 0.00200) | Beta(85.71, 52436.82) | CCDS (2015) |
| NL Female, 50-64 | 0.00525 | (0.00470, 0.00585) | Beta(318.57, 60361.00) | CCDS (2015) |
| NL Female, 65-79 | 0.01713 | (0.01572, 0.01864) | Beta(519.76, 29822.32) | CCDS (2015) |
| NL Female, 80+ | 0.02036 | (0.01757, 0.02347) | Beta(179.24, 8624.39) | CCDS (2015) |
| NL Male, 20-34 | 0.00042 | (0.00026, 0.00065) | Beta(17.81, 42395.24) | CCDS (2015) |
| NL Male, 35-49 | 0.00279 | (0.00236, 0.00327) | Beta(144.04, 51482.35) | CCDS (2015) |
| NL Male, 50-64 | 0.00926 | (0.00849, 0.01007) | Beta(522.92, 55947.49) | CCDS (2015) |
| NL Male, 65-79 | 0.01972 | (0.01815, 0.02139) | Beta(558.00, 27737.90) | CCDS (2015) |
| NL Male, 80+ | 0.03113 | (0.02649, 0.03634) | Beta(148.67, 4627.18) | CCDS (2015) |
| **Stroke** |  |  |  |  |
| BC Female, 20-34 | 0.00029 | (0.00024, 0.00034) | Beta(129.19, 445366.17) | CCDS (2015) |
| BC Female, 35-49 | 0.00076 | (0.00068, 0.00084) | Beta(346.44, 455495.85) | CCDS (2015) |
| BC Female, 50-64 | 0.00221 | (0.00209, 0.00234) | Beta(1198.16, 540955.92) | CCDS (2015) |
| BC Female, 65-79 | 0.00718 | (0.00688, 0.00748) | Beta(2184.68, 302088.14) | CCDS (2015) |
| BC Female, 80+ | 0.02165 | (0.02082, 0.02251) | Beta(2467.20, 111491.36) | CCDS (2015) |
| BC Male, 20-34 | 0.00024 | (0.00020, 0.00029) | Beta(109.25, 455081.22) | CCDS (2015) |
| BC Male, 35-49 | 0.00090 | (0.00082, 0.00099) | Beta(430.30, 477676.43) | CCDS (2015) |
| BC Male, 50-64 | 0.00317 | (0.00302, 0.00333) | Beta(1601.72, 503673.78) | CCDS (2015) |
| BC Male, 65-79 | 0.00994 | (0.00958, 0.01031) | Beta(2820.71, 280953.39) | CCDS (2015) |
| BC Male, 80+ | 0.02454 | (0.02349, 0.02563) | Beta(1971.05, 78348.83) | CCDS (2015) |
| NL Female, 35-49 | 0.00054 | (0.00036, 0.00077) | Beta(26.64, 49308.32) | CCDS (2015) |
| NL Female, 50-64 | 0.00169 | (0.00139, 0.00203) | Beta(106.97, 63186.29) | CCDS (2015) |
| NL Female, 65-79 | 0.00573 | (0.00501, 0.00652) | Beta(220.00, 38174.22) | CCDS (2015) |
| NL Female, 80+ | 0.01890 | (0.01654, 0.02151) | Beta(218.00, 11316.43) | CCDS (2015) |
| NL Male, 20-34 | 0.00021 | (0.00010, 0.00039) | Beta(8.06, 38353.21) | CCDS (2015) |
| NL Male, 35-49 | 0.00055 | (0.00037, 0.00078) | Beta(27.64, 50220.45) | CCDS (2015) |
| NL Male, 50-64 | 0.00282 | (0.00242, 0.00326) | Beta(172.69, 61066.45) | CCDS (2015) |
| NL Male, 65-79 | 0.00875 | (0.00783, 0.00974) | Beta(319.66, 36213.28) | CCDS (2015) |
| NL Male, 80+ | 0.01911 | (0.01617, 0.02242) | Beta(140.89, 7231.92) | CCDS (2015) |

CI: confidence intervals

CCDS: Canadian Chronic Disease Surveillance System

Heart disease: ischemic heart disease
