# Supplementary material for: Is it worth it? Cost-effectiveness analysis of a commercial physical activity app
Source: BMC Public Health. 2021 Oct 27;21:1950. doi: 10.1186/s12889-021-11988-y (PMC8548862; doi:10.1186/s12889-021-11988-y)
Supplement: Supplementary file 5 — Additional file 5. Age-, gender-, and disease-dependent mortality rates by health state. [file 12889_2021_11988_MOESM5_ESM.docx]

**Additional File 5.** Age-, gender-, and disease-dependent mortality rates by health state.

| **Mortality** | | | | |
| --- | --- | --- | --- | --- |
|  | Expected Value | 95% CI | Distribution | Reference |
| **Healthy** |  |  |  |  |
| Female, 13-19 | 0.0012 | (0.0011, 0.0013) | Beta(552.53, 459885.28) | Statistics Canada (2016) |
| Female, 20-34 | 0.0025 | (0.0023, 0.0026) | Beta(1064.44, 424711.89) | Statistics Canada (2016) |
| Female, 35-49 | 0.0054 | (0.0052, 0.0056) | Beta(2785.40, 513029.08) | Statistics Canada (2016) |
| Female, 50-64 | 0.0203 | (0.0199, 0.0207) | Beta(9693.41, 467814.31) | Statistics Canada (2016) |
| Female, 65-79 | 0.0733 | (0.0724, 0.0742) | Beta(23614.18, 298543.81) | Statistics Canada (2016) |
| Female, 80+ | 0.4124 | (0.4098, 0.4151) | Beta(54668.38, 77893.16) | Statistics Canada (2016) |
| Male, 13-19 | 0.0020 | (0.0019, 0.0022) | Beta(681.58, 340110.02) | Statistics Canada (2016) |
| Male, 20-34 | 0.0060 | (0.0057, 0.0062) | Beta(2199.48, 364380.36) | Statistics Canada (2016) |
| Male, 35-49 | 0.0095 | (0.0093, 0.0098) | Beta(5494.56, 572880.37) | Statistics Canada (2016) |
| Male, 50-64 | 0.0307 | (0.0302, 0.0311) | Beta(17330.91, 547193.77) | Statistics Canada (2016) |
| Male, 65-79 | 0.1079 | (0.1068, 0.1090) | Beta(32974.77, 272630.15) | Statistics Canada (2016) |
| Male, 80+ | 0.4980 | (0.4946, 0.5013) | Beta(42616.70, 42959.01) | Statistics Canada (2016) |
| **Breast Cancer** |  |  |  |  |
| *Female, 13-19* | *0.0012* | *(0.0011, 0.0013)* | *Beta(552.53, 459885.28)* | *Statistics Canada (2016)* |
| Female, 20-34 | 0.0026 | (0.0024, 0.0027) | Beta(1151.18, 441611.85) | Statistics Canada (2016) |
| Female, 35-49 | 0.0063 | (0.0060, 0.0065) | Beta(2424.19, 382368.52) | Statistics Canada (2016) |
| Female, 50-64 | 0.0228 | (0.0224, 0.0232) | Beta(12196.76, 522748.88) | Statistics Canada (2016) |
| Female, 65-79 | 0.0787 | (0.0777, 0.0796) | Beta(24289.25, 284341.60) | Statistics Canada (2016) |
| Female, 80+ | 0.4249 | (0.4223, 0.4276) | Beta(56798.12, 76875.97) | Statistics Canada (2016) |
| *Male, 13-19* | *0.0020* | *(0.0019, 0.0022)* | *Beta(681.58, 340110.02)* | *Statistics Canada (2016)* |
| *Male, 20-34* | *0.0060* | *(0.0058, 0.0062)* | *Beta(3436.69, 569344.87)* | *Statistics Canada (2016)* |
| *Male, 35-49* | *0.0095* | *(0.0093, 0.0098)* | *Beta(5494.56, 572880.37)* | *Statistics Canada (2016)* |
| Male, 50-64 | 0.0307 | (0.0302, 0.0311) | Beta(17330.91, 547193.77) | Statistics Canada (2016) |
| Male, 65-79 | 0.1080 | (0.1069, 0.1091) | Beta(33032.22, 272821.64) | Statistics Canada (2016) |
| Male, 80+ | 0.4981 | (0.4948, 0.5015) | Beta(42625.33, 42950.52) | Statistics Canada (2016) |
| **Colon Cancer** |  |  |  |  |
| *Female, 13-19* | *0.0012* | *(0.0011, 0.0013)* | *Beta(552.53, 459885.28)* | *Statistics Canada (2016)* |
| Female, 20-34 | 0.0025 | (0.0024, 0.0026) | Beta(2395.00, 955603.00) | Statistics Canada (2016) |
| Female, 35-49 | 0.0057 | (0.0055, 0.0059) | Beta(3102.55, 541204.11) | Statistics Canada (2016) |
| Female, 50-64 | 0.0215 | (0.0211, 0.0219) | Beta(10859.98, 494255.40) | Statistics Canada (2016) |
| Female, 65-79 | 0.0773 | (0.0765, 0.0783) | Beta(26148.41, 312123.43) | Statistics Canada (2016) |
| Female, 80+ | 0.4266 | (0.4240, 0.4293) | Beta(57084.28, 76727.91) | Statistics Canada (2016) |
| Male, 13-19 | 0.0020 | (0.0019, 0.0022) | Beta(681.58, 340110.02) | Statistics Canada (2016) |
| Male, 20-34 | 0.0060 | (0.0058, 0.0062) | Beta(3436.69, 569344.87) | Statistics Canada (2016) |
| Male, 35-49 | 0.0099 | (0.0096, 0.0101) | Beta(5964.59, 596519.60) | Statistics Canada (2016) |
| Male, 50-64 | 0.0325 | (0.0320, 0.0329) | Beta(19386.71, 577127.40) | Statistics Canada (2016) |
| Male, 65-79 | 0.1141 | (0.1131, 0.1153) | Beta(36616.89, 284302.36) | Statistics Canada (2016) |
| Male, 80+ | 0.5166 | (0.5132, 0.5199) | Beta(44160.39, 41322.36) | Statistics Canada (2016) |
| **Diabetes** |  |  |  |  |
| Female, 13-19 | 0.0012 | (0.0011, 0.0014) | Beta(245.57, 204392.90) | Statistics Canada (2016) |
| Female, 20-34 | 0.0025 | (0.0024, 0.0026) | Beta(2395.00, 955603.00) | Statistics Canada (2016) |
| Female, 35-49 | 0.0056 | (0.0054, 0.0058) | Beta(2994.94, 531816.24) | Statistics Canada (2016) |
| Female, 50-64 | 0.0209 | (0.0205, 0.0213) | Beta(10268.59, 481051.60) | Statistics Canada (2016) |
| Female, 65-79 | 0.0761 | (0.0752, 0.0770) | Beta(25375.82, 308077.81) | Statistics Canada (2016) |
| Female, 80+ | 0.4259 | (0.4232, 0.4285) | Beta(56966.56, 76789.15) | Statistics Canada (2016) |
| Male, 13-19 | 0.0020 | (0.0019, 0.0022) | Beta(681.58, 340110.02) | Statistics Canada (2016) |
| Male, 20-34 | 0.0060 | (0.0058, 0.0062) | Beta(3436.69, 569344.87) | Statistics Canada (2016) |
| Male, 35-49 | 0.0098 | (0.0095, 0.0101) | Beta(4059.23, 410147.91) | Statistics Canada (2016) |
| Male, 50-64 | 0.0318 | (0.0314, 0.0323) | Beta(18574.01, 565514.37) | Statistics Canada (2016) |
| Male, 65-79 | 0.1127 | (0.1115, 0.1138) | Beta(32736.58, 257738.83) | Statistics Canada (2016) |
| Male, 80+ | 0.5147 | (0.5113, 0.5180) | Beta(44008.45, 41494.66) | Statistics Canada (2016) |
| **Heart Disease** |  |  |  |  |
| *Female, 13-19* | *0.0012* | *(0.0011, 0.0013)* | *Beta(552.53, 459885.28)* | *Statistics Canada (2016)* |
| Female, 20-34 | 0.0025 | (0.0024, 0.0026) | Beta(2395.00, 955603.00) | Statistics Canada (2016) |
| Female, 35-49 | 0.0057 | (0.0055, 0.0059) | Beta(3102.55, 541204.11) | Statistics Canada (2016) |
| Female, 50-64 | 0.0221 | (0.0217, 0.0225) | Beta(11467.54, 507425.73) | Statistics Canada (2016) |
| Female, 65-79 | 0.0825 | (0.0816, 0.0835) | Beta(26581.38, 295617.19) | Statistics Canada (2016) |
| Female, 80+ | 0.4833 | (0.4807, 0.4860) | Beta(66022.09, 70584.76) | Statistics Canada (2016) |
| *Male, 13-19* | *0.0020* | *(0.0019, 0.0022)* | *Beta(681.58, 340110.02)* | *Statistics Canada (2016)* |
| Male, 20-34 | 0.0060 | (0.0058, 0.0062) | Beta(3436.69, 569344.87) | Statistics Canada (2016) |
| Male, 35-49 | 0.0106 | (0.0103, 0.0109) | Beta(4745.18, 442912.91) | Statistics Canada (2016) |
| Male, 50-64 | 0.0373 | (0.0368, 0.0378) | Beta(20581.64, 531204.96) | Statistics Canada (2016) |
| Male, 65-79 | 0.1295 | (0.1283, 0.1306) | Beta(42405.62, 285050.93) | Statistics Canada (2016) |
| Male, 80+ | 0.5956 | (0.5923, 0.5988) | Beta(52174.82, 35425.62) | Statistics Canada (2016) |
| **Stroke** |  |  |  |  |
| *Female, 13-19* | *0.0012* | *(0.0011, 0.0014)* | *Beta(245.57, 204392.90)* | *Statistics Canada (2016)* |
| Female, 20-34 | 0.0025 | (0.0024, 0.0026) | Beta(2395.00, 955603.00) | Statistics Canada (2016) |
| Female, 35-49 | 0.0056 | (0.0054, 0.0059) | Beta(1916.76, 340362.04) | Statistics Canada (2016) |
| Female, 50-64 | 0.0210 | (0.0207, 0.0214) | Beta(13539.31, 631189.93) | Statistics Canada (2016) |
| Female, 65-79 | 0.0779 | (0.0769, 0.0788) | Beta(23818.61, 281940.21) | Statistics Canada (2016) |
| Female, 80+ | 0.4558 | (0.4531, 0.4584) | Beta(61847.82, 73842.87) | Statistics Canada (2016) |
| *Male, 13-19* | *0.0020* | *(0.0019, 0.0022)* | *Beta(681.58, 340110.02)* | *Statistics Canada (2016)* |
| Male, 20-34 | 0.0060 | (0.0058, 0.0062) | Beta(3436.69, 569344.87) | Statistics Canada (2016) |
| Male, 35-49 | 0.0097 | (0.0095, 0.0100) | Beta(5727.19, 584704.83) | Statistics Canada (2016) |
| Male, 50-64 | 0.0318 | (0.0313, 0.0322) | Beta(18574.01, 565514.37) | Statistics Canada (2016) |
| Male, 65-79 | 0.1137 | (0.1126, 0.1148) | Beta(36377.02, 283561.57) | Statistics Canada (2016) |
| Male, 80+ | 0.5351 | (0.5318, 0.5385) | Beta(45566.63, 39588.72) | Statistics Canada (2016) |

CI: confidence intervals

*Italicized parameters not used as individuals were not in this state.
