# Supplementary material for: Is it worth it? Cost-effectiveness analysis of a commercial physical activity app
Source: BMC Public Health. 2021 Oct 27;21:1950. doi: 10.1186/s12889-021-11988-y (PMC8548862; doi:10.1186/s12889-021-11988-y)
Supplement: Supplementary file 6 — Additional file 6. Relative risk by engagement level, age group, and gender. [file 12889_2021_11988_MOESM6_ESM.docx]

**Additional File 6.** Relative risk for each chronic condition by engagement level, age group, and gender.

| **Relative Risk** | | | | |
| --- | --- | --- | --- | --- |
|  | Expected Value | 95% CI | Distribution | Reference |
| **Breast Cancer** |  |  |  |  |
| Regular User, Young | 0.94 | (0.90, 0.99) | Lognormal(-0.062, 0.024) | Rockhill et al. (1999) |
| Regular User, Old | 0.97 | (0.94, 1.02) | Lognormal(-0.031, 0.021) | McTiernan et al. (2003) |
| Committed User, Young | 0.88 | (0.80, 0.98) | Lognormal(-0.129, 0.052) | Rockhill et al. (199) |
| Committed User, Old | 0.95 | (0.88, 1.04) | Lognormal(-0.052, 0.043) | McTiernan et al. (2003) |
| **Colon Cancer** |  |  |  |  |
| Regular User, Female, Young | 0.92 | (0.87, 0.99) | Lognormal(-0.084, 0.033) | Mai et al. (2007) |
| Regular User, Female, Old | 0.99 | (0.97, 1.02) | Lognormal(-0.010, 0.013) | Howard et al. (2008) |
| Regular User, Male, Young | 0.93 | (0.89, 1.01) | Lognormal(-0.073, 0.032) | Friedenreich et al. (2006) |
| Regular User, Male, Old | 0.98 | (0.97, 1.00) | Lognormal(-0.020, 0.008) | Howard et al. (2008) |
| Committed User, Female, Young | 0.85 | (0.75, 0.97) | Lognormal(-0.165, 0.066) | Mai et al. (2007) |
| Committed User, Female, Old | 0.99 | (0.95, 1.04) | Lognormal(-0.010, 0.023) | Howard et al. (2008) |
| Committed User, Male, Young | 0.87 | (0.78, 1.03) | Lognormal(-0.142, 0.071) | Friedenreich et al. (2006) |
| Committed User, Male, Old | 0.97 | (0.94, 1.01) | Lognormal(-0.031, 0.018) | Howard et al. (2008) |
| **Diabetes** |  |  |  |  |
| Regular User, Female, Young | 0.90 | (0.75, 1.10) | Lognormal(-0.110, 0.098) | Manson et al. (1991) |
| Regular User, Female, Old | 0.97 | (0.93, 1.01) | Lognormal(-0.031, 0.021) | Folsom et al. (2000) |
| Regular User, Male, Young | 0.91 | (0.84, 1.00) | Lognormal(-0.095, 0.044) | Okada et al. (2000) |
| Regular User, Male, Old | 0.96 | (0.94, 0.99) | Lognormal(-0.041, 0.013) | Hu et al. (2001) |
| Committed User, Female, Young | 0.75 | (0.61, 0.90) | Lognormal(-0.293, 0.099) | Manson et al. (1991) |
| Committed User, Female, Old | 0.93 | (0.86, 1.01) | Lognormal(-0.073, 0.041) | Folsom et al. (2000) |
| Committed User, Male, Young | 0.82 | (0.68, 0.99) | Lognormal(-0.203, 0.096) | Okada et al. (2000) |
| Committed User, Male, Old | 0.93 | (0.89, 0.98) | Lognormal(-0.073, 0.025) | Hu et al. (2001) |
| **Heart Disease** |  |  |  |  |
| Regular User, Female, Young | 0.76 | (0.63, 1.08) | Lognormal(-0.284, 0.137) | Sundquist et al. (2005) |
| Regular User, Female, Old | 1.00 | (1.00, 1.00) | Lognormal(0.000, 0.003) | Armstrong et al. (2015) |
| Regular User, Male, Young | 0.76 | (0.63, 1.08) | Lognormal(-0.284, 0.137) | Sundquist et al. (2005) |
| Regular User, Male, Old | 0.99 | (0.97, 1.01) | Lognormal(-0.010, 0.010) | Tanasecu et al. (2002) |
| Committed User, Female, Young | 0.52 | (0.27, 1.17) | Lognormal(-0.724, 0.374) | Sundquist et al. (2005) |
| Committed User, Female, Old | 0.99 | (0.99, 1.00) | Lognormal(-0.010, 0.003) | Armstrong et al. (2015) |
| Committed User, Male, Young | 0.52 | (0.27, 1.17) | Lognormal(-0.724, 0.374) | Sundquist et al. (2005) |
| Committed User, Male, Old | 0.97 | (0.94, 1.02) | Lognormal(-0.031, 0.021) | Tanasecu et al. (2002) |
| **Stroke** |  |  |  |  |
| Regular User, Female, Young | 0.92 | (0.76, 1.15) | Lognormal(-0.089, 0.106) | Myint et al. (2006) |
| Regular User, Female, Old | 0.85 | (0.76, 0.96) | Lognormal(-0.164, 0.060) | Armstrong et al (2015) |
| Regular User, Male, Young | 0.91 | (0.70, 1.20) | Lognormal(-0.104, 0.137) | Myint et al. (2006) |
| Regular User, Male, Old | 0.87 | (0.75, 1.01) | Lognormal(-0.142, 0.076) | Calling et al. (2006) |
| Committed User, Female, Young | 0.93 | (0.79, 1.13) | Lognormal(-0.077, 0.091) | Myint et al. (2006) |
| Committed User, Female, Old | 0.70 | (0.53, 0.91) | Lognormal(-0.366, 0.138) | Armstrong et al (2015) |
| Committed User, Male, Young | 0.96 | (0.77, 1.19) | Lognormal(-0.047, 0.111) | Myint et al. (2006) |
| Committed User, Male, Old | 0.74 | (0.51, 1.02) | Lognormal(-0.317, 0.177) | Calling et al. (2006) |

CI: confidence intervals

Heart disease: ischemic heart disease only

Regular: users engaged for 24 to 51 weeks

Committed: users engaged for 52 weeks

Young: ages 13 to 49 years

Old: ages 50 to 79 years
