# Supplementary material for: Is it worth it? Cost-effectiveness analysis of a commercial physical activity app
Source: BMC Public Health. 2021 Oct 27;21:1950. doi: 10.1186/s12889-021-11988-y (PMC8548862; doi:10.1186/s12889-021-11988-y)
Supplement: Supplementary file 7 — Additional file 7. Age-, gender-, and geography dependent average annual medical costs by chronic disease. [file 12889_2021_11988_MOESM7_ESM.docx]

**Additional File 7.** Age-, gender-, and geography dependent average annual medical costs by chronic disease.

| **Cost of Disease State** | | | | |
| --- | --- | --- | --- | --- |
|  | Expected Value | 95% CI | Distribution | Reference |
| **Breast Cancer** |  |  |  |  |
| *BC, 13-19* | *1,397.60* | *(1257.84, 1537.36)* | *Gamma(384.16, 0.27)* | *EBIC** |
| BC, 20-34 | 1,397.60 | (1,257.84, 1,537.36) | Gamma(384.16, 0.27) | EBIC* |
| BC, 35-49 | 3,207.13 | (2,886.42, 3,527.84) | Gamma(384.16, 0.12) | EBIC* |
| BC, 50-64 | 2,881.92 | (2,593.73, 3,170.11) | Gamma(384.16, 0.13) | EBIC* |
| BC, 65-79 | 2,410.99 | (2,169.89, 2,652.09) | Gamma(384.16, 0.16) | EBIC* |
| BC, 80+ | 1,062.45 | (956.21, 1168.70) | Gamma(384.16, 0.36) | EBIC* |
| *NL, 13-19* | *1,610.31* | *(1,044.28, 1,276.34)* | *Gamma(384.16, 0.33)* | *EBIC** |
| NL, 20-34 | 1,160.31 | (1,044.28, 1,276.34) | Gamma(384.16, 0.33) | EBIC* |
| NL, 35-49 | 3,748.56 | (3,373.70, 4,123.42) | Gamma(384.16, 0.10) | EBIC* |
| NL, 50-64 | 3,343.64 | (3,009.28, 3,678.00) | Gamma(384.16, 0.11) | EBIC* |
| NL, 65-79 | 3,825.81 | (3,443.23, 4,208.39) | Gamma(384.16, 0.10) | EBIC* |
| NL, 80+ | 4,113.93 | (3,702.54, 4,525.32) | Gamma(384.16, 0.09) | EBIC* |
| **Colon Cancer** |  |  |  |  |
| *BC Female, 13-19* | *4,437.00* | *(3,993.30, 4,880.70)* | *Gamma(384.16, 0.09)* | *EBIC** |
| BC Female, 20-34 | 4,437.00 | (3,993.30, 4,880.70) | Gamma(384.16, 0.09) | EBIC* |
| BC Female, 35-49 | 8,458.45 | (7,612.61, 9,304.30) | Gamma(384.16, 0.05) | EBIC* |
| BC Female, 50-64 | 10,253.22 | (9,227.90, 11,278.54) | Gamma(384.16, 0.04) | EBIC* |
| BC Female, 65-79 | 10,087.89 | (9,079.10, 11,096.68) | Gamma(384.16, 0.04) | EBIC* |
| BC Female, 80+ | 7,983.09 | (7,184.78, 8,781.40) | Gamma(384.16, 0.05) | EBIC* |
| BC Male, 13-19 | 2,979.10 | (2,681.19, 3,277.01) | Gamma(384.16, 0.13) | EBIC* |
| BC Male, 20-34 | 2,979.10 | (2,681.19, 3,277.01) | Gamma(384.16, 0.13) | EBIC* |
| BC Male, 35-49 | 9,748.34 | (8,773.51, 10,723.17) | Gamma(384.16, 0.04) | EBIC* |
| BC Male, 50-64 | 9,962.03 | (8,965.83, 10,958.23) | Gamma(384.16, 0.04) | EBIC* |
| BC Male, 65-79 | 9,802.51 | (8,822.26, 10,782.76) | Gamma(384.16, 0.04) | EBIC* |
| BC Male, 80+ | 9,358.90 | (8,423.01, 10,294.79) | Gamma(384.16, 0.04) | EBIC* |
| *NL Female, 13-19* | *4,955.56* | *(4,460.00, 5,451.12)* | *Gamma(384.16, 0.08)* | *EBIC** |
| *NL Female, 20-34* | *4,955.56* | *(4,460.00, 5,451.12)* | *Gamma(384.16, 0.08)* | *EBIC** |
| NL Female, 35-49 | 13,969.98 | (12,572.98, 15,366.98) | Gamma(384.16, 0.03) | EBIC* |
| NL Female, 50-64 | 16,032.67 | (14,429.40, 17,635.94) | Gamma(384.16, 0.02) | EBIC* |
| NL Female, 65-79 | 11,896.88 | (10,707.19, 13,086.57) | Gamma(384.16, 0.03) | EBIC* |
| NL Female, 80+ | 10,598.59 | (9,538.73, 11,658.45) | Gamma(384.16, 0.04) | EBIC* |
| *NL Male, 13-19* | *7,241.59* | *(6,517.43, 7,965.75)* | *Gamma(384.16, 0.05)* | *EBIC** |
| *NL Male, 20-34* | *7,241.59* | *(6,517.43, 7,965.75)* | *Gamma(384.16, 0.05)* | *EBIC** |
| NL Male, 35-49 | 13,361.78 | (12,025.60, 14,697.96) | Gamma(384.16, 0.03) | EBIC* |
| NL Male, 50-64 | 17,983.77 | (16,185.39, 19,782.15) | Gamma(384.16, 0.02) | EBIC* |
| NL Male, 65-79 | 15,875.15 | (14,287.64, 17,462.67) | Gamma(384.16, 0.02) | EBIC* |
| NL Male, 80+ | 10,580.96 | (9,522.86, 11,639.06) | Gamma(384.16, 0.04) | EBIC* |
| **Diabetes** |  |  |  |  |
| BC Female, 13-19 | 1,003.58 | (903.22, 1,103.94) | Gamma(384.16, 0.38) | EBIC* |
| BC Female, 20-34 | 1,003.58 | (903.22, 1,103.94) | Gamma(384.16, 0.38) | EBIC* |
| BC Female, 35-49 | 1,023.76 | (921.38, 1,126.14) | Gamma(384.16, 0.38) | EBIC* |
| BC Female, 50-64 | 1,427.09 | (1,284.38, 1,569.80) | Gamma(384.16, 0.27) | EBIC* |
| BC Female, 65-79 | 937.32 | (843.59, 1,031.05) | Gamma(384.16, 0.41) | EBIC* |
| BC Female, 80+ | 905.80 | (815.22, 996.38) | Gamma(384.16, 0.42) | EBIC* |
| BC Male, 13-19 | 702.83 | (632.55, 773.11) | Gamma(384.16, 0.55) | EBIC* |
| BC Male, 20-34 | 702.83 | (632.55, 773.11) | Gamma(384.16, 0.55) | EBIC* |
| BC Male, 35-49 | 779.04 | (701.14, 856.94) | Gamma(384.16, 0.49) | EBIC* |
| BC Male, 50-64 | 1,159.23 | (1,043.31, 1,275.15) | Gamma(384.16, 0.33) | EBIC* |
| BC Male, 65-79 | 803.89 | (723.50, 884.28) | Gamma(384.16, 0.48) | EBIC* |
| BC Male, 80+ | 1,061.98 | (955.78, 1,168.18) | Gamma(384.16, 0.36) | EBIC* |
| NL Female, 13-19 | 997.40 | (897.66, 1,097.14) | Gamma(384.16, 0.39) | EBIC* |
| NL Female, 20-34 | 997.40 | (897.66, 1,097.14) | Gamma(384.16, 0.39) | EBIC* |
| NL Female, 35-49 | 1,744.24 | (1,569.82, 1,918.66) | Gamma(384.16, 0.22) | EBIC* |
| NL Female, 50-64 | 853.77 | (768.39, 939.15) | Gamma(384.16, 0.45) | EBIC* |
| NL Female, 65-79 | 888.92 | (800.03, 977.81) | Gamma(384.16, 0.43) | EBIC* |
| NL Female, 80+ | 791.77 | (712.59, 870.95) | Gamma(384.16, 0.49) | EBIC* |
| NL Male, 13-19 | 718.17 | (646.35, 789.99) | Gamma(384.16, 0.53) | EBIC* |
| NL Male, 20-34 | 718.17 | (646.35, 789.99) | Gamma(384.16, 0.53) | EBIC* |
| NL Male, 35-49 | 1,044.26 | (939.83, 1,148.69) | Gamma(384.16, 0.37) | EBIC* |
| NL Male, 50-64 | 1,313.21 | (1,181.89, 1,444.53) | Gamma(384.16, 0.29) | EBIC* |
| NL Male, 65-79 | 977.56 | (879.80, 1,075.32) | Gamma(384.16, 0.39) | EBIC* |
| NL Male, 80+ | 730.62 | (657.56, 803.68) | Gamma(384.16, 0.53) | EBIC* |
| **Heart Disease** |  |  |  |  |
| *BC Female, 13-19* | *337.57* | *(303.81, 371.33)* | *Gamma(384.16, 1.14)* | *EBIC** |
| BC Female, 20-34 | 337.57 | (303.81, 371.33) | Gamma(384.16, 1.14) | EBIC* |
| BC Female, 35-49 | 1,043.75 | (939.38, 1,148.13) | Gamma(384.16, 0.37) | EBIC* |
| BC Female, 50-64 | 1,196.72 | (1,077.05, 1,316.39) | Gamma(384.16, 0.32) | EBIC* |
| BC Female, 65-79 | 939.03 | (845.13, 1,032.93) | Gamma(384.16, 0.41) | EBIC* |
| BC Female, 80+ | 681.78 | (613.60, 749.96) | Gamma(384.16, 0.56) | EBIC* |
| *BC Male, 13-19* | *460.47* | *(414.42, 506.52)* | *Gamma(384.16, 0.83)* | *EBIC** |
| BC Male, 20-34 | 460.47 | (414.42, 506.52) | Gamma(384.16, 0.83) | EBIC* |
| BC Male, 35-49 | 2,475.03 | (2,227.53, 2,722.53) | Gamma(384.16, 0.16) | EBIC* |
| BC Male, 50-64 | 2,681.30 | (2,413.17, 2,949.43) | Gamma(384.16, 0.14) | EBIC* |
| BC Male, 65-79 | 1,441.52 | (1,297.37, 1,585.67) | Gamma(384.16, 0.27) | EBIC* |
| BC Male, 80+ | 1,200.19 | (1,080.17, 1,320.21) | Gamma(384.16, 0.32) | EBIC* |
| *NL Female, 13-19* | *13.75* | *(12.38, 15.13)* | *Gamma(384.16, 27.94)* | *EBIC** |
| NL Female, 20-34 | 13.75 | (12.38, 15.13) | Gamma(384.16, 27.94) | EBIC* |
| NL Female, 35-49 | 1,452.93 | (1,307.64, 1,598.22) | Gamma(384.16, 0.26) | EBIC* |
| NL Female, 50-64 | 1,581.12 | (1,423.01, 1,739.23) | Gamma(384.16, 0.24) | EBIC* |
| NL Female, 65-79 | 2,488.16 | (2,239.34, 2,736.98) | Gamma(384.16, 0.15) | EBIC* |
| NL Female, 80+ | 1,046.11 | (941.50, 1,150.72) | Gamma(384.16, 0.37) | EBIC* |
| *NL Male, 13-19* | *112.70* | *(101.43, 123.97)* | *Gamma(384.16, 3.41)* | *EBIC** |
| NL Male, 20-34 | 112.70 | (101.43, 123.97) | Gamma(384.16, 3.41) | EBIC* |
| NL Male, 35-49 | 2,511.51 | (2,260.36, 2,762.66) | Gamma(384.16, 0.15) | EBIC* |
| NL Male, 50-64 | 2,126.19 | (1,913.57, 2,338.81) | Gamma(384.16, 0.18) | EBIC* |
| NL Male, 65-79 | 1,586.10 | (1,427.49, 1,744.71) | Gamma(384.16, 0.24) | EBIC* |
| NL Male, 80+ | 1,255.16 | (1,129.64, 1,380.68) | Gamma(384.16, 0.31) | EBIC* |
| **Stroke** |  |  |  |  |
| *BC Female, 13-19* | *183.34* | *(165.01, 201.67)* | *Gamma(384.16, 2.1)* | *EBIC** |
| BC Female, 20-34 | 183.34 | (165.01, 201.67) | Gamma(384.16, 2.1) | EBIC* |
| BC Female, 35-49 | 356.68 | (321.01, 392.35) | Gamma(384.16, 1.08) | EBIC* |
| BC Female, 50-64 | 482.01 | (433.81, 530.21) | Gamma(384.16, 0.80) | EBIC* |
| BC Female, 65-79 | 1,251.66 | (1,126.49, 1,376.83) | Gamma(384.16, 0.31) | EBIC* |
| BC Female, 80+ | 1,575.17 | (1,417.65, 1,732.69) | Gamma(384.16, 0.24) | EBIC* |
| *BC Male, 13-19* | *146.81* | *(132.13, 161.49)* | *Gamma(384.16, 2.62)* | *EBIC** |
| BC Male, 20-34 | 146.81 | (132.13, 161.49) | Gamma(384.16, 2.62) | EBIC* |
| BC Male, 35-49 | 1,024.78 | (922.30, 1,127.26) | Gamma(384.16, 0.37) | EBIC* |
| BC Male, 50-64 | 1,612.44 | (1,451.20, 1,773.68) | Gamma(384.16, 0.24) | EBIC* |
| BC Male, 65-79 | 1,319.97 | (1,187.97, 1,451.97) | Gamma(384.16, 0.29) | EBIC* |
| BC Male, 80+ | 1,319.14 | (1,187.23, 1,451.05) | Gamma(384.16, 0.29) | EBIC* |
| *NL Female, 13-19* | *190.46* | *(171.41, 209.51)* | *Gamma(384.16, 2.02)* | *EBIC** |
| NL Female, 20-34 | 190.46 | (171.41, 209.51) | Gamma(384.16, 2.02) | EBIC* |
| NL Female, 35-49 | 926.03 | (833.43, 1,018.63) | Gamma(384.16, 0.41) | EBIC* |
| NL Female, 50-64 | 389.99 | (350.99, 428.99) | Gamma(384.16, 0.99) | EBIC* |
| NL Female, 65-79 | 250.76 | (225.68, 275.84) | Gamma(384.16, 1.53) | EBIC* |
| NL Female, 80+ | 415.39 | (373.85, 456.93) | Gamma(384.16, 0.92) | EBIC* |
| *NL Male, 13-19* | *7.99* | *(7.19, 8.79)* | *Gamma(384.16, 48.08)* | *EBIC** |
| NL Male, 20-34 | 7.99 | (7.19, 8.79) | Gamma(384.16, 48.08) | EBIC* |
| NL Male, 35-49 | 504.81 | (454.33, 555.29) | Gamma(384.16, 0.76) | EBIC* |
| NL Male, 50-64 | 561.55 | (505.40, 617.71) | Gamma(384.16, 0.68) | EBIC* |
| NL Male, 65-79 | 391.34 | (352.21, 430.47) | Gamma(384.16, 0.98) | EBIC* |
| NL Male, 80+ | 364.06 | (327.65, 400.47) | Gamma(384.16, 1.06) | EBIC* |

*Standard error not reported in these datasets and boundaries of 10% were assumed for the lower and upper bounds; italicized parameters not used as individuals were not in this state.

CI: confidence intervals

BC: British Columbia

NL: Newfoundland Labrador

EBIC: Economic Burden of Illness in Canada
