# Supplementary material for: Is it worth it? Cost-effectiveness analysis of a commercial physical activity app
Source: BMC Public Health. 2021 Oct 27;21:1950. doi: 10.1186/s12889-021-11988-y (PMC8548862; doi:10.1186/s12889-021-11988-y)
Supplement: Supplementary file 8 — Additional file 8. Age-, gender-, and geography-dependent average annual intervention costs by engagement level. [file 12889_2021_11988_MOESM8_ESM.docx]

**Additional File 8.** Age-, gender-, and geography-dependent average annual intervention costs by engagement level.

| **Carrot Cost** | | | | |
| --- | --- | --- | --- | --- |
|  | Expected Value (CAD $) | 95% CI | Distribution | Reference |
| **Limited User Costs** |  |  |  |  |
| BC Female, 13-19 | 1.11 | (0.98, 1.24) | Gamma(291.38, 262.61) | Carrot App |
| BC Female, 20-34 | 1.13 | (1.08, 1.19) | Gamma(1795.85, 1584.30) | Carrot App |
| BC Female, 35-49 | 1.09 | (1.02, 1.16) | Gamma(884.31, 813.53) | Carrot App |
| BC Female, 50-64 | 1.05 | (0.94, 1.16) | Gamma(344.47, 329.32) | Carrot App |
| BC Female, 65+ | 1.04 | (0.74, 1.33) | Gamma(47.40, 45.73) | Carrot App |
| BC Male, 13-19 | 1.17 | (1.01, 1.34) | Gamma(200.81, 170.93) | Carrot App |
| BC Male, 20-34 | 1.21 | (1.14, 1.28) | Gamma(1128.56, 935.20) | Carrot App |
| BC Male, 35-49 | 1.14 | (1.04, 1.24) | Gamma(468.50, 411.58) | Carrot App |
| BC Male, 50-64 | 1.06 | (0.90, 1.22) | Gamma(161.37, 152.21) | Carrot App |
| BC Male, 65+ | 1.01 | (0.68, 1.34) | Gamma(35.53, 35.25) | Carrot App |
| NL Female, 13-19 | 1.17 | (0.96, 1.39) | Gamma(110.96, 94.56) | Carrot App |
| NL Female, 20-34 | 1.11 | (1.04, 1.18) | Gamma(980.29, 885.22) | Carrot App |
| NL Female, 35-49 | 1.03 | (0.94, 1.13) | Gamma(481.12, 464.93) | Carrot App |
| NL Female, 50-64 | 1.04 | (0.88, 1.21) | Gamma(157.97, 151.33) | Carrot App |
| NL Female, 65+ | 0.85 | (0.77, 0.94) | Gamma(384.16, 451.95) | Carrot App* |
| NL Male, 13-19 | 1.18 | (0.90, 1.47) | Gamma(65.56, 55.41) | Carrot App |
| NL Male, 20-34 | 1.19 | (1.07, 1.31) | Gamma(384.78, 322.36) | Carrot App |
| NL Male, 35-49 | 1.13 | (0.97, 1.30) | Gamma(181.90, 160.74) | Carrot App |
| NL Male, 50-64 | 1.14 | (0.82, 1.46) | Gamma(47.68, 41.85) | Carrot App |
| NL Male, 65+ | 0.98 | (0.88, 1.08) | Gamma(384.16, 392.00) | Carrot App* |
| **Occasional User Costs** |  |  |  |  |
| BC Female, 13-19 | 2.44 | (2.23, 2.65) | Gamma(511.22, 209.49) | Carrot App |
| BC Female, 20-34 | 2.35 | (2.27, 2.43) | Gamma(2984.31, 1270.06) | Carrot App |
| BC Female, 35-49 | 2.25 | (2.13, 2.37) | Gamma(1387.08, 616.70) | Carrot App |
| BC Female, 50-64 | 2.24 | (2.04, 2.44) | Gamma(490.53, 219.13) | Carrot App |
| BC Female, 65+ | 2.45 | (1.58, 3.31) | Gamma(30.92, 12.64) | Carrot App |
| BC Male, 13-19 | 2.53 | (2.24, 2.83) | Gamma(289.88, 114.38) | Carrot App |
| BC Male, 20-34 | 2.63 | (2.51, 2.75) | Gamma(1799.43, 684.39) | Carrot App |
| BC Male, 35-49 | 2.57 | (2.39, 2.76) | Gamma(722.96, 280.92) | Carrot App |
| BC Male, 50-64 | 2.38 | (2.08, 2.68) | Gamma(236.81, 99.58) | Carrot App |
| BC Male, 65+ | 3.29 | (2.13, 4.45) | Gamma(30.96, 9.40) | Carrot App |
| NL Female, 13-19 | 2.35 | (2.05, 2.65) | Gamma(235.72, 100.31) | Carrot App |
| NL Female, 20-34 | 2.09 | (1.98, 2.19) | Gamma(1454.51, 697.20) | Carrot App |
| NL Female, 35-49 | 2.04 | (1.89, 2.19) | Gamma(725.68, 356.15) | Carrot App |
| NL Female, 50-64 | 1.82 | (1.56, 2.09) | Gamma(177.59, 97.31) | Carrot App |
| NL Female, 65+ | 1.96 | (0.49, 3.42) | Gamma(6.83, 3.49) | Carrot App |
| NL Male, 13-19 | 2.30 | (1.79, 2.81) | Gamma(78.11, 33.97) | Carrot App |
| NL Male, 20-34 | 2.25 | (2.07, 2.42) | Gamma(638.12, 283.81) | Carrot App |
| NL Male, 35-49 | 2.09 | (1.82, 2.35) | Gamma(239.26, 114.54) | Carrot App |
| NL Male, 50-64 | 1.93 | (1.53, 2.32) | Gamma(91.96, 47.71) | Carrot App |
| NL Male, 65+ | 2.28 | (0.62, 3.94) | Gamma(7.29, 3.20) | Carrot App |
| **Regular User Costs** |  |  |  |  |
| BC Female, 13-19 | 5.18 | (4.90, 5.46) | Gamma(1341.51, 259.07) | Carrot App |
| BC Female, 20-34 | 5.22 | (5.12, 5.32) | Gamma(10002.98, 1917.12) | Carrot App |
| BC Female, 35-49 | 5.34 | (5.18, 5.50) | Gamma(4323.19, 809.97) | Carrot App |
| BC Female, 50-64 | 5.76 | (5.47, 6.05) | Gamma(1526.12, 265.09) | Carrot App |
| BC Female, 65+ | 5.87 | (4.86, 6.88) | Gamma(129.79, 22.11) | Carrot App |
| BC Male, 13-19 | 5.57 | (5.14, 6.00) | Gamma(643.36, 115.58) | Carrot App |
| BC Male, 20-34 | 5.55 | (5.41, 5.68) | Gamma(6357.41, 1146.08) | Carrot App |
| BC Male, 35-49 | 5.95 | (5.74, 6.17) | Gamma(2921.95, 490.84) | Carrot App |
| BC Male, 50-64 | 6.45 | (5.96, 6.94) | Gamma(660.65, 102.44) | Carrot App |
| BC Male, 65+ | 5.91 | (4.84, 6.98) | Gamma(116.90, 19.79) | Carrot App |
| NL Female, 13-19 | 4.57 | (4.20, 4.93) | Gamma(608.61, 133.30) | Carrot App |
| NL Female, 20-34 | 4.65 | (4.51, 4.80) | Gamma(3954.40, 850.39) | Carrot App |
| NL Female, 35-49 | 4.68 | (4.46, 4.90) | Gamma(1717.14, 366.91) | Carrot App |
| NL Female, 50-64 | 5.23 | (4.80, 5.65) | Gamma(588.78, 112.67) | Carrot App |
| NL Female, 65+ | 5.26 | (2.71, 7.80) | Gamma(16.41, 3.12) | Carrot App |
| NL Male, 13-19 | 4.73 | (3.94, 5.52) | Gamma(136.57, 28.87) | Carrot App |
| NL Male, 20-34 | 4.90 | (4.65, 5.15) | Gamma(1434.75, 292.78) | Carrot App |
| NL Male, 35-49 | 5.11 | (4.72, 5.50) | Gamma(666.29, 130.40) | Carrot App |
| NL Male, 50-64 | 5.93 | (5.15, 6.72) | Gamma(221.72, 37.36) | Carrot App |
| NL Male, 65+ | 7.00 | (4.82, 9.18) | Gamma(39.61, 5.66) | Carrot App |
| **Committed User Costs** |  |  |  |  |
| BC Female, 13-19 | 7.19 | (6.70, 7.69) | Gamma(813.26, 113.08) | Carrot App |
| BC Female, 20-34 | 7.63 | (7.46, 7.79) | Gamma(8168.39, 1071.16) | Carrot App |
| BC Female, 35-49 | 8.54 | (8.29, 8.80) | Gamma(4390.18, 513.85) | Carrot App |
| BC Female, 50-64 | 9.85 | (9.33, 10.37) | Gamma(1369.38, 139.06) | Carrot App |
| BC Female, 65-79 | 10.63 | (8.85, 12.40) | Gamma(137.34, 12.92) | Carrot App |
| BC Female, 80+ | 8.14 | (7.33, 8.95) | Gamma(384.16, 47.19) | Carrot App* |
| BC Male, 13-19 | 7.20 | (6.49, 7.92) | Gamma(389.24, 54.04) | Carrot App |
| BC Male, 20-34 | 7.88 | (7.65, 8.11) | Gamma(4610.03, 585.04) | Carrot App |
| BC Male, 35-49 | 8.59 | (8.28, 8.90) | Gamma(2944.38, 342.73) | Carrot App |
| BC Male, 50-64 | 9.83 | (9.23, 10.44) | Gamma(1014.16, 103.17) | Carrot App |
| BC Male, 65+ | 9.81 | (7.85, 11.77) | Gamma(96.38, 9.82) | Carrot App |
| NL Female, 13-19 | 6.57 | (6.03, 7.11) | Gamma(576.42, 87.72) | Carrot App |
| NL Female, 20-34 | 6.97 | (6.70, 7.24) | Gamma(2520.50, 361.74) | Carrot App |
| NL Female, 35-49 | 8.01 | (7.61, 8.41) | Gamma(1536.30, 191.85) | Carrot App |
| NL Female, 50-64 | 8.62 | (7.89, 9.36) | Gamma(526.90, 61.12) | Carrot App |
| NL Female, 65+ | 7.35 | (4.83, 9.88) | Gamma(32.55, 4.43) | Carrot App |
| NL Male, 13-19 | 5.99 | (4.87, 7.12) | Gamma(108.61, 18.12) | Carrot App |
| NL Male, 20-34 | 6.92 | (6.57, 7.28) | Gamma(1469.83, 212.31) | Carrot App |
| NL Male, 35-49 | 7.14 | (6.64, 7.64) | Gamma(773.98, 108.41) | Carrot App |
| NL Male, 50-64 | 7.66 | (6.60, 8.72) | Gamma(200.26, 26.16) | Carrot App |
| NL Male, 65+ | 6.98 | (4.27, 9.68) | Gamma(25.53, 3.66) | Carrot App |

CI: confidence intervals

BC: British Columbia

NL: Newfoundland & Labrador

Limited: users engaged for fewer than 12 weeks

Occasional: users engaged for 12 to 23 weeks

Regular: users engaged for 24 to 51 weeks

Committed: users engaged for 52 weeks
