# Supplementary material for: Is it worth it? Cost-effectiveness analysis of a commercial physical activity app
Source: BMC Public Health. 2021 Oct 27;21:1950. doi: 10.1186/s12889-021-11988-y (PMC8548862; doi:10.1186/s12889-021-11988-y)
Supplement: Supplementary file 9 — Additional file 9. Age-, gender-, and geography dependent average annual medical costs by chronic disease. [file 12889_2021_11988_MOESM9_ESM.docx]

**Additional File 9.** Age-, gender-, and geography dependent average annual medical costs by chronic disease.

| **Utility** | | | | |
| --- | --- | --- | --- | --- |
|  | Expected Value | 95% CI | Distribution | Reference |
| Healthy | 0.95 | (0.86, 1.00) | Beta(18.26, 0.96) | Bansback et al., (2012)  ** |
| Breast Cancer | 0.69 | (0.66, 0.72) | Beta(499.13, 223.55) | Yousefi et al. (2016) |
| Colon Cancer | 0.81 | (0.79, 0.83) | Beta(1197.97, 274.73) | Färkkilä et al. (2013) |
| Diabetes | 0.76 | (0.74, 0.78) | Beta(2048.87, 646.25) | Tsiplova et al. (2016) |
| Heart Disease | 0.75 | (0.73, 0.77) | Beta(1631.34, 543.03) | Tsiplova et al. (2016) |
| Stroke | 0.59 | (0.52, 0.65) | Beta(133.27, 92.02) | Golicki et al. (2015) |

CI: confidence intervals

**standard error not reported, lower boundary of 10% was used and an upper boundary of 1.00 was used; italicized parameters not used as individuals were not in this state
