# Supplementary material for: Is it worth it? Cost-effectiveness analysis of a commercial physical activity app
Source: BMC Public Health. 2021 Oct 27;21:1950. doi: 10.1186/s12889-021-11988-y (PMC8548862; doi:10.1186/s12889-021-11988-y)
Supplement: Supplementary file 10 — Additional file 10. Lower and upper bounds for each parameter in the deterministic sensitivity analysis. [file 12889_2021_11988_MOESM10_ESM.docx]

**Additional File 10.** Lower and upper bounds for each parameter in the deterministic sensitivity analysis.

| Parameter^a^ | Lower Bound Cost | Upper Bound Cost |
| --- | --- | --- |
| Diabetes Relative Risk | 7,112.18 | 20,015.08 |
| Health State Utility | 18,943.5 | 9,040.62 |
| Heart Disease Relative Risk | 9,869.64 | 16,651.81 |
| Stroke Relative Risk | 8,773.70 | 1,5491.36 |
| Stroke Risk | 13,267.09 | 7,399.39 |
| Colon Cancer Risk | 12,286.23 | 7,167.07 |
| Heart Disease Risk | 11,229.41 | 6,315.99 |
| Mortality | 11,800.03 | 7,010.60 |
| Breast Cancer Risk | 11,342.04 | 7,365.41 |
| Diabetes Risk | 12,000.76 | 9,037.40 |
| Disease States Utility | 9,871.80 | 12,524.12 |
| Carrot Registration Cost | 10,366.4 | 12,262.39 |
| Breast Cancer Relative Risk | 9,734.55 | 11,196.46 |
| Carrot Start Cost  (0.00, 1.00) | 10,550.69 | 11,488.40 |
| Colon Cancer Relative Risk | 10,619.39 | 11,137.72 |
| Colon Cancer Cost | 11,072.52 | 11,494.56 |
| Discount Rate  (0.00, 0.03) | 11,319.67 | 10,913.25 |
| Diabetes Cost | 11,316.29 | 10,910.34 |
| Heart Disease Cost | 11,308.00 | 10,918.63 |
| Stroke Cost | 11,162.67 | 11,063.96 |
| Breast Cancer Cost | 11,154.11 | 11,072.52 |

^a^For discount rate and Carrot start costs, a single parameter was varied and the lower and upper bounds of the parameter are found in the Table. For all other parameters, a set of parameters was varied to the lower and upper bounds of the confidence interval limits as displayed in Additional File 10. For relative risk measures, where confidence intervals exceeded 1.00, relative risks were bounded to a maximum upper bound of 1.00.
